# Effectiveness of Autologous Hematopoietic Stem Cell Transplantation versus Alemtuzumab and Ocrelizumab in Relapsing Multiple Sclerosis: A Single Center Cohort Study

**DOI:** 10.1002/ana.27247

**Published:** 2025-04-19

**Authors:** Paolo Antonio Muraro, Antonio Zito, Alessio Signori, Maria Pia Sormani, Eleonora Rigoni, Federica Pollidoro, Roberto Bergamaschi, Alice Mariottini, Omar Malik, Ashwini Nandoskar, Victoria Singh‐Curry, Varun Mehra, Majid Kazmi, Ian Gabriel, Eli Silber, Richard Nicholas, Antonio Scalfari

**Affiliations:** ^1^ Department of Brain Sciences, Faculty of Medicine Imperial College London London UK; ^2^ Imperial College Healthcare NHS Trust London UK; ^3^ Multiple Sclerosis Center, IRCCS Mondino Foundation Pavia Italy; ^4^ Department of Health Sciences University of Genoa Genoa Italy; ^5^ Scientific Institute for research, hospitalisation, and Healthcare, University hospital San Martino Genoa Italy; ^6^ Department of Neurosciences University of Florence, Careggi Hospital Florence Italy; ^7^ Cromwell, Hospital London UK; ^8^ Department of Hematological Medicine King's College Hospital NHS Trust London UK; ^9^ Center of Hematology, Faculty of Medicine Imperial College Healthcare Trust London UK; ^10^ Department of Neurology Kings College Hospital NHS Foundation Trust London UK

## Abstract

**Objective:**

To compare clinical and radiological outcomes among relapsing multiple sclerosis patients treated with autologous hematopoietic stem cell transplantation (AHSCT), alemtuzumab (ATZ), and ocrelizumab (OCR).

**Methods:**

From a London (UK) hospital‐based observational cohort, modeled data were obtained from 621 relapsing–remitting multiple sclerosis patients, who were treated with AHSCT (n = 103), ATZ (n = 204), and OCR (n = 314), and were followed up for 43, 43, and 32 median months, respectively. The annualized relapse rate, new magnetic resonance imaging (MRI) lesions, and disability progression on Expanded Disability Status Scale were assessed.

**Results:**

AHSCT showed superior efficacy compared with ATZ and OCR. After 5‐year follow up, the mean annualized relapse rate (0.026 vs 0.087; *p* < 0.001), the risk of relapses (HR 0.29, 95% CI 0.13–0.63; *p* = 0.002), and of MRI activity (HR 0.33, 95% CI 0.15–0.72; *p* = 0.006) were significantly lower in AHSCT versus ATZ group. Compared with OCR, after 3‐year follow‐up AHSCT showed a significantly lower annualized relapse rate (0.028 vs 0.073; *p* = 0.02) and a trend to reduced risk of relapse (HR 0.45, 95% CI 0.18–1.14; *p* = 0.09), but similar low rates (6%) of new MRI activity (HR 0.86, 95% CI 0.28–2.67; *p* = 0.80). In addition, there was a similar risk of Expanded Disability Status Scale progression in AHSCT, and both ATZ (HR 1.19, 95% CI 0.71–2.00; *p* = 0.50) and OCR (HR 1.08, 95% CI 0.57–2.04; *p* = 0.82) groups.

**Interpretation:**

AHSCT was followed by greater prevention of relapses compared with ATZ and OCR, and suppressed more profoundly MRI activity than ATZ, but similarly to OCR, albeit with shorter follow up. The risk of accumulating disability was similar among the treated groups. Studies with larger sample sizes and longer follow up may enable confirmation of these findings or detection of any additional differential effects. ANN NEUROL 2025;98:294–307

Multiple sclerosis (MS) is a chronic immune‐mediated disease of the central nervous system, which can cause severe physical and cognitive disability and inflammatory and neurodegenerative mechanisms are considered its 2 major pathological components.^1^ The suppression of inflammatory disease activity with disease‐modifying treatments (DMTs) represents the cornerstone of MS therapeutic management, although the sustained long‐term disease remission remains often elusive,^2^ and safety concerns in the context of long term drug exposure represents an important limitation. Autologous hematopoietic stem cell transplantation (AHSCT) has gained increasing interest as a treatment option, particularly for MS patients with limited response to DMTs. The procedure aims to remove all adaptive immunity, which in MS includes pathogenetic autoreactive lymphocytes, promoting a non‐inflammatory immune reconstitution.^3^ The refinement of patients selection toward earlier stages of MS, the evolution of AHSCT protocols, and clinical experience have contributed to improve its efficacy, to substantially reduce treatment‐related mortality and to improve management of adverse events.^4^ The European Bone Marrow Transplantation Society guidelines are now recommending AHSCT as an option for treating patients with aggressive forms of MS, even without prior exposure to DMTs.^5^

After AHSCT, in most MS patients, remission of inflammatory parameters (relapses and magnetic resonance imaging [MRI] activity), prevention of disability worsening, and long‐term disease stabilization have been reported.^6^, ^7^, ^8^, ^9^, ^10^, ^11^, ^12^, ^13^ Although AHSCT is believed to exert higher efficacy than standard treatments,^8^ real‐world evidence of its effectiveness, compared with highly active treatments, remains scarce. Previous observational studies were carried out on small cohorts enriched with patients presenting with severe disease features.^11^, ^14^, ^15^ In addition, 2 randomized controlled trials showed superiority of AHSCT, but their small size and the choice of DMT comparators represent important limitations. One study compared AHSCT against mitoxantrone,^16^ which is no longer in use because of its toxicity. The other trial compared AHSCT with a treatment arm of heterogenous DMTs, including highly efficacious and first‐line treatments.^17^ A recent analysis of the MSBase registry demonstrated better outcomes after AHSCT compared with fingolimod and natalizumab treatment, but similar clinical outcomes when compared with patients receiving ocrelizumab (OCR) infusions. Limitations of the study included different AHSCT protocols across centers, the short follow up of the OCR cohort, and the lack of MRI data.^18^ We previously reported the efficacy and safety outcomes in a cohort of MS patients, who were selected for AHSCT under homogenous criteria, and had their procedure and were followed up at 2 collaborating London centers.^19^ The dataset was recently expanded, and by using the overlap weighting propensity score method (PSOW),^20^ we set out to perform comparative analyses among patients receiving AHSCT in the real world, and 2 highly active DMTs, alemtuzumab (ATZ)^21^ and OCR.^22^

## Methods

Data were retrospectively collected from a consecutive series of patients, with a diagnosis of relapsing–remitting (RR) MS, who were followed up at the MS tertiary center of Imperial College NHS Trust (Charing Cross Hospital, London, UK), where they were routinely clinically evaluated by 5 neurologists expert in MS, and where they had regular MRI assessments. Patients included in the analysis received at least 2 courses of ATZ (cycle 1: 12 mg i.v. daily for 5 days, cycle 2: 12 mg for 3 days) from July 2010 to November 2021 or were treated with OCR (2 300‐mg i.v. doses initially administered 14 days apart, followed by 600 mg every 6 months) from January 2017 to November 2021. The AHSCT was carried out initially at the hematology center of King's College Hospital between February 2012 and November 2021, and subsequently also of Hammersmith Hospital from April 2016 to January 2020, both in London (UK). Once approved for AHSCT, patients were assigned to 1 of the 2 hematology centers based on the residence address, referring clinicians, and waiting times. Ethical approval from research committee and consent from patients was not required, as data were collected anonymized. The dataset was locked for analysis in November 2021.

The indication for AHSCT was initially (2012–2015) based on the agreement of at least 2 neurologists and 1 hematologist with expertise in AHSCT that the treatment was in the patient's best interest. In September 2015, AHSCT eligibility criteria (Table 1) were formally defined to select patients with clinically and radiologically active forms of RRMS, refractory to standard DMT, and each case's eligibility had to be reviewed and approved by the Pan‐London multidisciplinary team panel of neurologists and hematologists. The multidisciplinary team panel assessed the eligibility based on clinical information collected on referral forms and on MRI results, which had previously been reviewed with the neuroradiologist. For some cases, who did not strictly meet inclusion criteria, but convincingly fulfilled the overall eligibility profile, AHSCT was offered, documenting the specific basis for the approval.

**Table 1:** Patients Eligibility Criteria for Treatment with Autologous Hematopoietic Stem Cell Transplantation

Inclusion criteria
Diagnosis of MS according to 2017 McDonald criteria
Age 18–65 years
Disease duration since diagnosis of ≤15 years
EDSS score between 0 and 6.5
MRI activity was defined by ≥1 gadolinium‐enhancing (>3 mm) lesion (off steroids for 1 month) or ≥2 new T2 lesions on MRI within the past 12 months.
Patients with RRMS had to experience treatment failure with at least one licensed DMT of high efficacy, defined as evidence of relapse or EDSS score increase and MRI activity, after being on treatment for at least 6 months
Exclusion criteria
Eligibility for an ethically approved clinical trial in which AHSCT is offered as 1 of the treatment arms
Inability to adequately understand risk and benefits of AHSCT, and give written informed consent
Prior treatment with total lymphoid irradiation and AHSCT or allogeneic hematopoietic stem cell transplantation

AHSCT = autologous hematopoietic stem cell transplantation; DMT, disease‐modifying treatment; EDSS = Expanded Disability Status Scale; MRI = magnetic resonance imaging; MS = multiple sclerosis; RRMS = relapsing–remitting multiple sclerosis.

### AHSCT Procedures

Patients underwent AHSCT according to the approved protocols at the 2 centers.^19^ At King's College Hospital, peripheral blood stems cells (PBSCs) were mobilized after administration of cyclophosphamide 4 g/m^2^ over 2 days (51 patients), or 2 g/m^2^ over 1 day (3 patients). After modification of the protocol in November 2018, PBSCs were mobilized with granulocyte colony‐stimulating factor (G‐CSF; 5 μg/kg SC) for 7 days until leukapheresis. Conditioning was performed with cyclophosphamide (50 mg/kg for 4 days) and rabbit anti‐thymocyte globulin (2.5 mg/kg/day for 3 days: total dose 7.5 mg/kg) for in vivo lymphodepletion followed by stem cells infusion. At Hammersmith Hospital, PBSCs were mobilized with cyclophosphamide 2 g/m^2^ (48 patients) and daily G‐CSF (5 μg/kg SC) starting from day +3 from cyclophosphamide until leukapheresis. For conditioning, cyclophosphamide (50 mg/kg for 4 days) and rabbit anti‐thymocyte globulin (2 or 2.5 mg/kg/day for 3 days: total dose 6–7.5 mg/kg) were used. After reinfusion of the autologous PBSC product, G‐CSF was administered starting from day 7 after AHSCT until engraftment. At Hammersmith Hospital, 1 patient was mobilized with cyclophosphamide 1 g/m^2^ followed by G‐CSF and plerixafor (2 doses), and conditioned with BEAM‐ATG due to intolerance of cyclophosphamide.

### Study Inclusion Criteria

We included patients who had a diagnosis of RRMS according to the 2017 McDonald criteria,^23^ and had a clinical and radiological follow up of at least 24 months after therapy initiation. Patients who received AHSCT or were treated with ATZ or OCR in the previous 2 years were excluded to avoid an overlap of therapeutic effects. As per standard clinical practice, follow‐up visits with Expanded Disability Status Scale (EDSS)^24^ evaluation and standard MRI assessments were performed at a minimum annually. Baseline was defined as the day of PBSC mobilization for AHSCT, or first ATZ or OCR dose. Patients were censored at discontinuing therapy, switching to another treatment or at last recorded assessment. The following data were extracted from the medical records: sex, age at disease onset and at treatment commencement, date of disease onset and disease duration at baseline, EDSS scores spanning from 2 years before baseline up to the end of the follow up, number of relapses and MRI activity spanning from 2 years before treatment initiation up to the final assessment, and treatment history (number of DMTs and low/high efficacy DMTs) prior to baseline.

### Outcomes

The primary outcomes of the study were on‐treatment annualized relapse rate (ARR), the cumulative hazard of relapses and of new MRI activity, the proportion of patients free from relapse and free from new brain MRI activity. A relapse was defined as the occurrence of new neurological symptoms, or worsening of pre‐existing symptoms, lasting at least 24 h, in the absence of associated fever or infections, and occurring ≥30 days from a previous relapse. Individual annualized relapse rates were calculated between the treatment onset and the last follow up. The MRI activity was defined as the occurrence of at least 1 new lesion in T2‐weighted scans, or as the occurrence of contrast enhancement in T1‐weighted images.

Secondary endpoints were the cumulative hazard incidence of disability accumulation, of disability improvement, and of “no evidence of disease activity” (NEDA) status failure. Disability accumulation was defined as an increase on the EDSS by 1 point (1.5 point if the baseline EDSS was 0, and 0.5 point if baseline EDSS was >5.5) confirmed after at least 6 months. Disability improvement was defined as a decrease in EDSS by 1 point (1.5 point if baseline EDSS was 1.5, and 0.5 point if baseline EDSS was >6) confirmed after at least 6 months. EDSS assessments were performed by the treating physicians, and scores recorded within 30 days of a previous relapse were excluded. NEDA was defined by the concomitant absence of clinical relapses, of EDSS worsening, and of new MRI activity.

Although comparison of safety outcomes was not an objective of this study, we report the adverse events observed following AHSCT, as its risk profile is recognized as a topic of interest.

### Statistical Analysis

To address potential confounding due to differences in baseline characteristics between the 3 treatment groups, we attempted to compute inverse weighting from propensity scores estimated via multinominal logistic regression.^20^ However, this approach did not allow proper balancing of all features among the 3 treatments groups. As an alternative, we ran 2 separate multivariable logistic regression models to calculate the probability (the propensity score) of each patient to receive AHSCT versus alemtuzumab or AHSCT versus ocrelizumab conditional on clinical and demographic characteristics. To further address this issue, we performed a sensitivity analysis in which weights were also estimated from generalized boosted model‐based propensity scores. Compared with treatments pair analyses performed independently, this approach reduces potential biases, as it allows to simultaneously consider all treatment groups within the same model for a more accurate balancing of covariates across the multiple categories (presented as supplemental material).^25^

Each multivariable logistic regression model included the following baseline covariates: sex, age, time elapsed since the onset of symptoms, EDSS score at baseline, EDSS changes (increase or decrease) in the year preceding the commencement of the treatment, the number of relapses experienced in the 2 years preceding the commencement of the treatment, MRI activity (new or enlarging T2 lesions; categorized as 0, 1–2, or ≥3 lesions) in the 2 years preceding the commencement of the treatment, prior treatment history (none, moderate, or high efficacy DMTs). Moderate efficacy treatments included interferon beta, Copaxone, fingolimod, and dimethyl fumarate, whereas high‐efficacy treatments included natalizumab, rituximab, cladribine, alemtuzumab (administered >2 years before study entry), mitoxantrone, and cyclophosphamide.^26^ Differences between the treatment groups before and after application of PSOW were assessed using the standardized mean difference; a standardized mean difference >0.10 was considered as an indication of relevant imbalance.

The survival time to endpoints was estimated using a weighted Kaplan–Meier (KM) method and compared by using the weighted Cox regression model to generate the hazard ratio (HR) and relative 95% confidence interval (CI), whereas a weighted negative binomial regression model was used to compare relapse rate, and results were reported as relapse rate ratio (ARR ratio). To assess the robustness to unmeasured confounders, we used the E‐values together with the corresponding 95% CI, as proposed by VanderWeele and Ding.^27^ The higher the E‐value, the stronger the association of the unknown confounder with treatment and outcome to nullify the observed effect should be; we computed the E‐value for the observed point estimate and for the 95% confidence limit closest to the null. To allow better interpretation of analyses results obtained from adjusted weighted data, we also performed KM analyses calculated from the unadjusted crude cohort (presented as supplemental material).

The post‐hoc power analysis of the comparison between AHSCT (n = 103) versus alemtuzumab (n = 204) showed a statistical power of 90%, and a level of significance α of 0.05. The minimum detectable treatment effect on relapse outcome (ARR ratio) was 0.72. With the same assumptions reported above, the minimum detectable treatment effect defined from the HR was 0.54 for time to first relapse, 0.50 for new MRI activity and for disability progression, 2.50 for disability improvement, and 0.61 for NEDA.

The post‐hoc power analysis of the comparison between AHSCT (n = 103) versus ocrelizumab (n = 314) showed a statistical power of 90%, and a level of significance α of 0.05. The minimum detectable treatment effect on relapse outcome (ARR ratio) was 0.74. With the same assumptions reported above, the minimum detectable treatment effect defined from the HR was 0.68 for time to first relapse, 0.59 for new MRI activity, 0.68 for disability progression, 1.50 for disability improvement, and 0.60 for NEDA.

Statistical analysis was performed using Stata (v.16; StataCorp, College Station, TX, USA).

## Results

**Figure 1:**
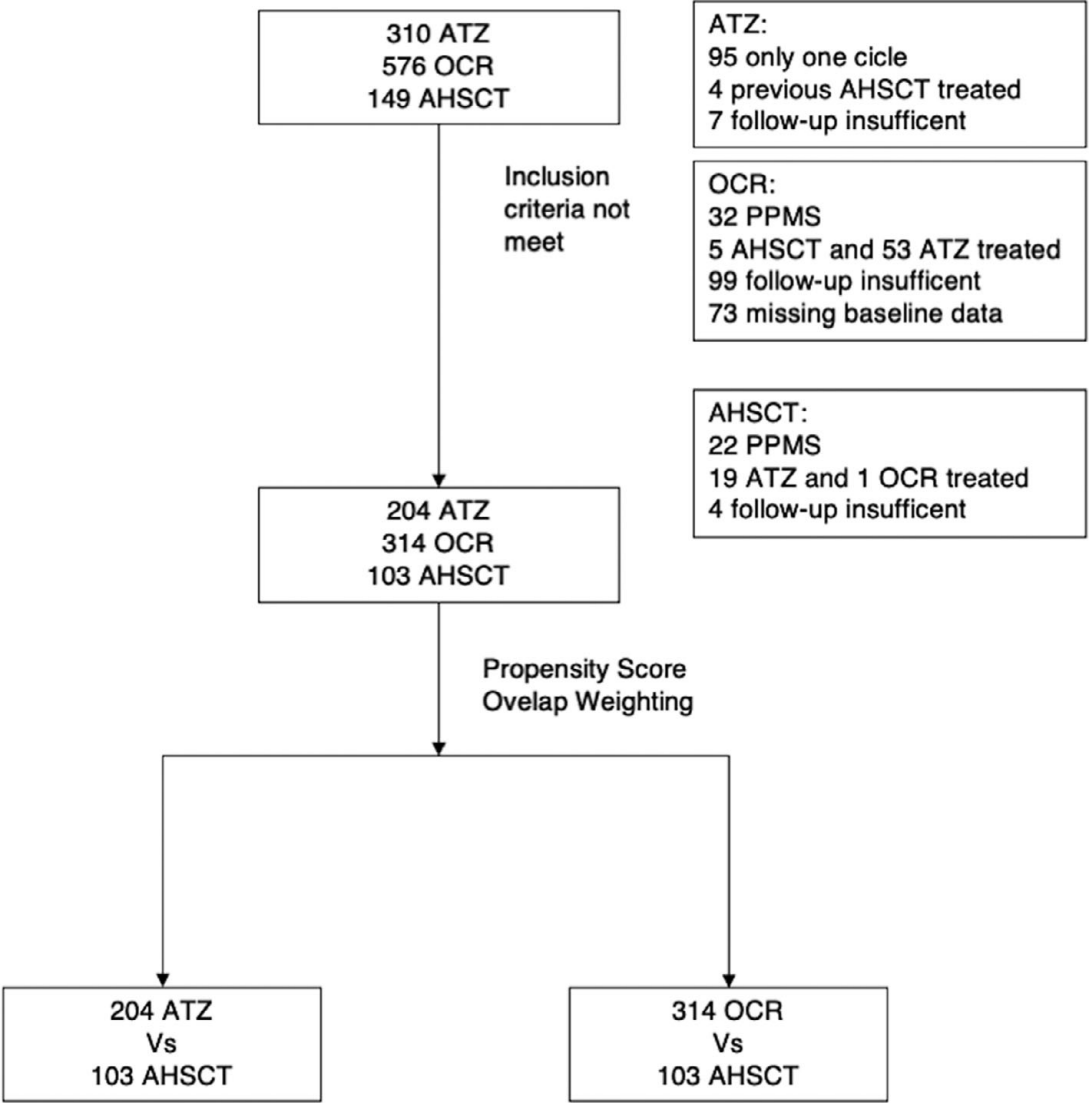
Flowchart showing patient selection based on inclusion and exclusion criteria. AHSCT = autologous hematopoietic stem cell transplantation; ATZ = alemtuzumab; OCR = ocrelizumab; PPMS = primary progressive multiple sclerosis.

Data on 1,035 RRMS patients, who were treated with AHSCT, ATZ, and OCR, were available, and 621 (AHSCT n = 103; ATZ n = 204; OCR n = 314) patients met the inclusion criteria (Fig 1). The cohort baseline demographics, and clinical and radiological characteristics before and after PSOW are shown in Tables 2 and 3. Before PSOW, at treatment initiation, the patients in the AHSCT group were older and had longer disease duration compared with the ATZ group, and they had more relapses and MRI activity in the preceding 2 years, and higher disability score, compared with both the ATZ and OCR groups. The median follow up was 43 months (interquartile range [IQR] 29–53), 43 months (IQR 35–58), and 32 months (IQR: 24–36) in the AHSCT, ATZ, and OCR cohorts, respectively. The clinical and radiological outcomes during the observation period of the 3 cohorts without PSOW are summarized in Table S1; 9.7%, 25.5%, and 15.7% had new relapses, 9.7%, 27.5%, and 4.8% had new MRI activity, and 31.1%, 28.9%, and 17.1% experienced EDSS progression, in the AHSCT, ATZ, and OCR group, respectively. Clinical and radiological features of patients experiencing relapses/MRI activity and EDSS progression while on treatment are outlined in Tables S2 and S3.

**Table 2:** Baseline Clinical and Radiological Features Before and After Propensity Score Overlap Weighting in the Hematopoietic Stem Cell Transplantation and Alemtuzumab Groups

Variables at baseline	HSCT before PSOW	ATZ before PSOW	SMD before PSOW	HSCT after PSOW	ATZ after PSOW	SMD after PSOW
N	103	204		103	204	
Sex, F/M (%)	57.3/42.7	66.2/33.8	0.18	62.3/37.7	62.3/37.7	0.000
Mean age, yr (SD)	42.3 (9.2)	42 (10.2)	0.029	43.4 (9.7)	43.4 (10.0)	0.000
Mean MS duration, yr (SD)	11.1 (6.0)	8.8 (6.6)	0.37	9.9 (5.7)	9.9 (7.1)	0.000
Median EDSS (25th–75th percentiles)	6 (5–6.5)	3 (2–6)	1.08	6 (3.5–6)	6 (3.5–6)	0.000
Mean EDSS change from previous 2 years (SD)	0.52 (1.07)	0.19 (0.68)	0.37	0.40 (0.97)	0.40 (0.95)	0.000
New MRI activity during 2 years before treatment	85.4%	69.1%	0.40	77.0%	77.0%	0.000
1 new T2 lesion	29.1%	34.3%	0.49	34.8%	34.8%	0.000
≥2 new T2 lesions	56.3%	34.8%		42.2%	42.2%	
ARR during 2 years before treatment, mean (SD)	0.61 (0.62)	0.56 (0.37)	0.11	0.57 (0.62)	0.57 (0.37)	0.000
Treatment naïve	6.8%	24.5%	0.48	14.0%	14.0%	0.000
Mean no. previous DMTs, (SD), median	2.09 (1.33), 2	1.05 (0.75), 1	0.96	1.31 (0.92), 1	1.31 (0.74), 1	0.000
Low‐/medium‐efficacy DMTs	28.2%	42.7%	0.68	34.1%	34.1%	0.000
High‐efficacy DMTs	65.0%	32.8%	0.68	51.9%	51.9%	0.000

Baseline was defined as the first day of hematopoietic stem cell transplantation (HSCT) conditioning, or first alemtuzumab (ATZ) cycle. High‐efficacy disease‐modifying treatments (DMTs): natalizumab, mitoxantrone, rituximab, alemtuzumab, and cladribine. Low‐efficacy DMTs: interferons, glatiramer acetate, dimethyl fumarate, and fingolimod.

ARR = annualized relapse rate; EDSS = Expanded Disability Status Scale; MRI = magnetic resonance imaging; MS = multiple sclerosis; PSOW = Propensity Score Overlap Weighting; SMD = standardized mean differences.

**Table 3:** Baseline Clinical and Radiological Features Before And After Propensity Score Overlap Weighting In The Hematopoietic Stem cell transplantation and Ocrelizumab Groups

Variables at baseline	HSCT before PSOW	OCR before PSOW	SMD before PSOW	HSCT after PSOW	OCR after PSOW	SMD after PSOW
N	103	314		103	314	
Sex, F/M (%)	57.3/42.7	66.9/33.1	0.20	63.9/36.1	63.9/36.1	0.000
Mean age, yr (SD)	42.3 (9.2)	45 (11.2)	0.26	44.2 (9.3)	44.2 (10.5)	0.000
Mean MS duration, yr (SD)	11.1 (6.0)	12 (8.7)	0.12	11.2 (6.3)	11.2 (6.9)	0.000
EDSS median (25th–75th percentiles)	6 (5–6.5)	4 (2–6)	0.84	6 (3.5–6)	6 (3.5–6.5)	0.000
Mean EDSS change from previous 2 years, (SD)	0.52 (1.07)	0.46 (1.16)	0.061	0.50 (1.06)	0.50 (1.16)	0.000
New MRI activity during 2 years before treatment	85.4%	43.9%	0.96	71.9%	71.9%	0.000
1 new T2 lesion	29.1%	18.2%	0.92	31.2%	31.2%	0.000
≥2 new T2 lesions	56.3%	25.2%		40.7%	40.7%	
Mean ARR during 2 years before treatment (SD)	0.61 (0.62)	0.46 (0.36)	0.30	0.52 (0.58)	0.52 (0.38)	0.000
Treatment naïve	6.8%	17.8%	0.34	13.7%	13.7%	0.000
Mean no. previous DMTs, (SD), median	2.09 (1.33), 2	1.27 (0.75), 1	0.76	1.44 (1.08), 1	1.44 (0.72), 2	0.000
Low‐/medium‐efficacy DMTs	28.2%	54.5%	0.65	37.6%	37.6%	0.000
High‐efficacy DMTs	65.0%	27.7%	0.65	48.7%	48.7%	0.000

Baseline was defined as the first day of hematopoietic stem cell transplantation (HSCT) conditioning or first Ocrelizumab dose. High‐efficacy disease‐modifying treatments (DMTs): natalizumab, mitoxantrone, rituximab, alemtuzumab, and cladribine. Low‐efficacy DMTs: interferons, glatiramer acetate, dimethyl fumarate, and fingolimod.

ARR = annualized relapse rate; EDSS = Expanded Disability Status Scale; MRI = magnetic resonance imaging; MS = multiple sclerosis; OCR = ocrelizumab; PSOW = Propensity Score Overlap Weighting; SMD = standardized mean differences.

### Comparison of HSCT versus ATZ

**Figure 2:**
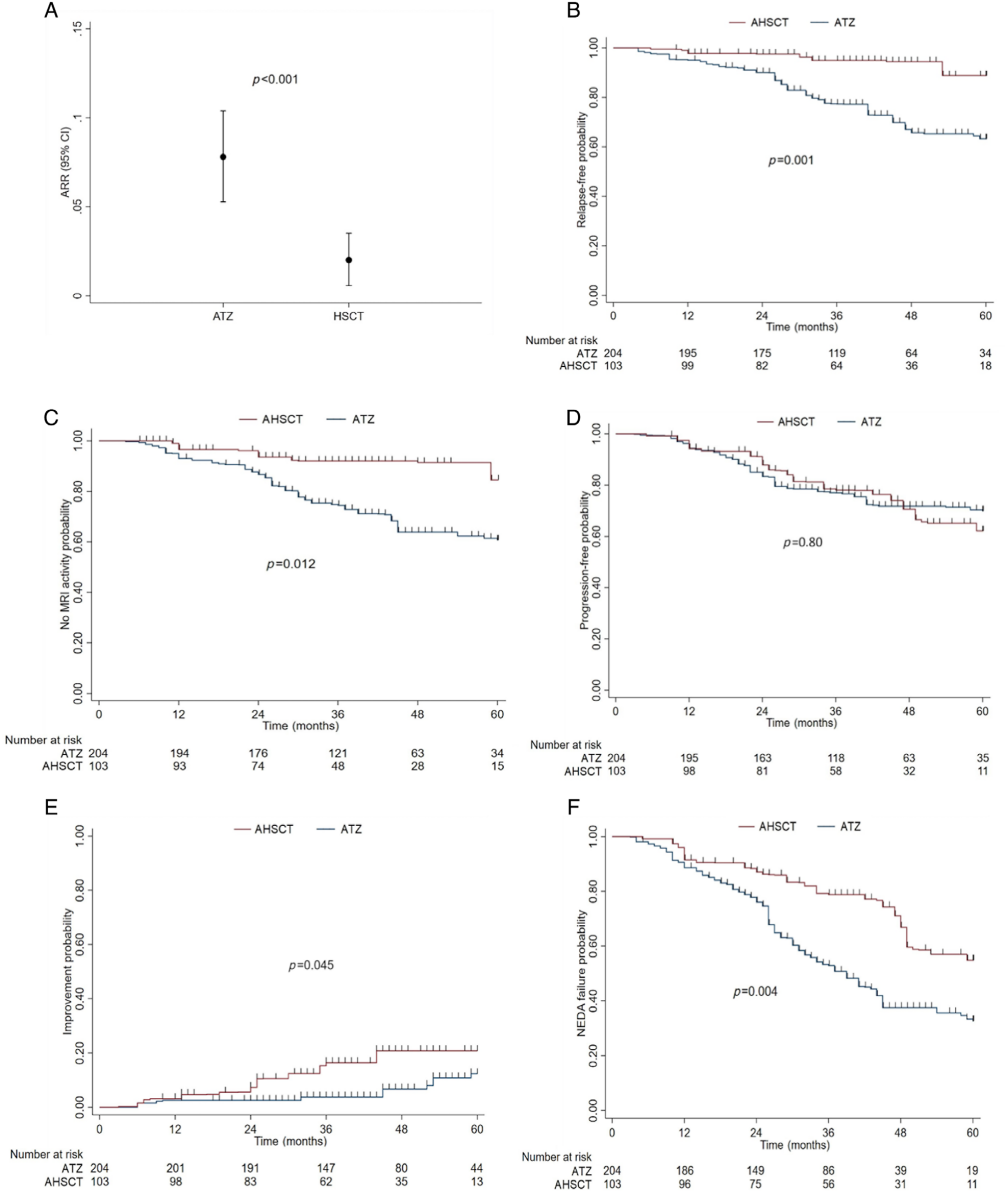
Comparative analysis of efficacy outcomes between the autologous hematopoietic stem cell transplantation (AHSCT) and alemtuzumab (ATZ) groups. (A) Annualized relapse ratio during the follow‐up period. (B) Weighted Kaplan–Meier analysis: cumulative probability of relapse. (C) Weighted Kaplan–Meier analysis: cumulative probability of new magnetic resonance imaging (MRI) activity. (D) Weighted Kaplan–Meier analysis: cumulative probability of Expanded Disability Status Scale progression. (E) Weighted Kaplan–Meier analysis: cumulative probability of Expanded Disability Status Scale improvement. (F) Weighted Kaplan–Meier analysis: cumulative probability of no evidence of disease activity (NEDA) failure. [Color figure can be viewed at www.annalsofneurology.org]

Table 2 shows the clinical and radiological baseline features in pairwise AHSCT‐ATZ cohorts, after PSOW. The ARR over 5 years was significantly lower among patients who received AHSCT compared with those who were treated with ATZ (ARR mean: 0.020 vs 0.078; ARR ratio: 0.26, 95% CI 0.12–0.57; *p* < 0.001; Fig 2A). In addition, being treated with AHSCT compared with ATZ was associated with a significantly lower weighted cumulative incidence of clinical relapses (HR 0.24, 95% CI 0.10–0.55; *p* = 0.001; Fig 2B). After 3 years, the weighted cumulative KM estimate of remaining free of relapses was 95% (95% CI 87–99) versus 77% (95% CI 71–82), whereas at 5 years, it was 89% (95% CI 75–96) versus 63% (95% CI 54–71), in the AHSCT and ATZ groups, respectively. The E‐value RR for unmeasured confounding was 7.2 for the ARR ratio (2.9 for its CI lower limit) and 7.8 (3.0 for its CI lower limit) for relapse‐free survival.

In line with the relapse analysis, the use of AHSCT compared with ATZ was associated with a significantly lower weighted risk of new MRI activity (HR 0.34, 95% CI 0.15–0.79; *p* = 0.012). The weighted cumulative KM estimate of remaining free of new MRI activity after 3 years of follow up was 92% (95% CI 86–98) in the AHSCT group and 74% (95% CI 69–80) in the ATZ group, and after 5 years this dropped to 85% and 71%, respectively (Fig 2C). The E‐value for unmeasured confounding was 5.3 (1.8 for its CI lower limit).

During the observation period, in the 2 weighted groups, patients had similar risk of experiencing confirmed disability accumulation (HR 0.92, 95% CI 0.50–1.71; *p* = 0.80; Fig 2D). Among AHSCT‐treated patients compared with ATZ, the weighted cumulative KM estimate of EDSS progression after 3 years was 22% (95% CI 13–31) and 23% (95% CI 17–29), whereas after 5 years it was 38% (95% CI 24–54) and 30% (95% CI 22–40), respectively. At the last follow up, the mean EDSS change from baseline was +0.068 (SD 1.10) for AHSCT patients, and +0.56 (SD 1.13) for those who were treated with ATZ, whereas the median EDSS change was of 0 (IQR 0–0.5) and 0 (IQR 0–1), respectively.

Treatment with AHSCT was associated with a significantly higher weighted probability of experiencing EDSS improvement (HR 2.68, 95% CI 1.02–7.00; *p* = 0.045; Fig 2E), which occurred in a larger proportion of AHSCT‐treated patients (19%) compared with ATZ‐treated patients (4%). The weighted cumulative KM estimate of disability improvement after 3 years, was 16% (95% CI 7–26) versus 4% (95% CI 2–6), and after 5 years it was 21% (95% CI 13–32) versus 12 (95% CI 8–21) in the AHSCT and ATZ groups, respectively.

Overall, a significantly smaller proportion of patients who received HSCT compared with ATZ (36.9% vs 52.0%) experienced NEDA status failure (HR 0.49, 95% CI 0.31–0.80; *p* = 0.004; Fig 2F). The weighted cumulative KM estimate of maintaining NEDA status at 3 years was 79% (95% CI 70–88) and 53% (95% CI 46–60), whereas at 5 years was 55% (95% CI 40–68) versus 33% (95% CI 24–42), in the AHSCT and ATZ groups, respectively. The E‐value RR for the unmeasured confounder of the NEDA estimate and its CI lower limit were 3.5 and 1.8, respectively.

### Comparison of AHSCT versus OCR

**Figure 3:**
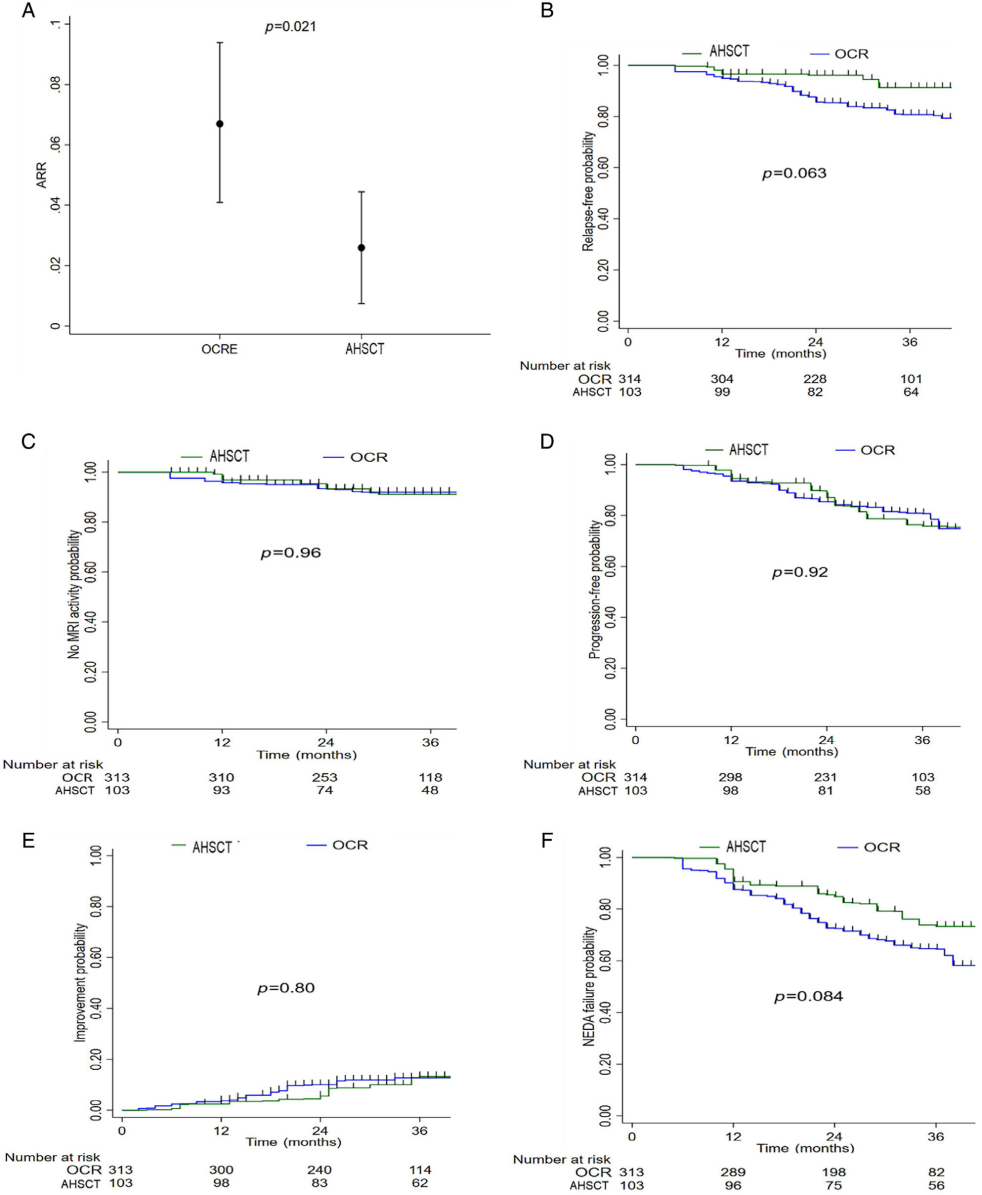
Comparative analysis of efficacy outcomes between the autologous hematopoietic stem cell transplantation (AHSCT) and ocrelizumab (OCR) groups. (A) Annualized relapse ratio during the follow‐up period. (B) Weighted Kaplan–Meier analysis: cumulative probability of relapse. (C) Weighted Kaplan–Meier analysis: cumulative probability of new magnetic resonance imaging (MRI) activity. (D) Weighted Kaplan–Meier analysis: cumulative probability of Expanded Disability Status Scale progression. (E) Weighted Kaplan–Meier analysis: cumulative probability of Expanded Disability Status Scale improvement; (F) Weighted Kaplan Meier analysis: cumulative probability of no evidence of disease activity (NEDA) failure. [Color figure can be viewed at www.annalsofneurology.org]

Table 3 shows clinical and radiological baseline features in pairwise AHSCT and OCR cohorts after PSOW. In the OCR group, patients received a mean of 5 (SD 1.5) infusions and were followed up for a shorter time (median 34 vs 43 months) compared with the AHSCT group. The use of AHSCT compared with OCR was associated with a significantly lower mean ARR (mean 0.026 vs 0.067; ARR ratio 0.38, 95% CI 0.17–0.87; *p* = 0.021; Fig 3A), and with a lower, albeit not significantly, weighted risk of clinical relapse (HR 0.40, 95% CI 0.15–1.05; *p* = 0.063; Fig 3B). After 3 years from baseline, the weighted cumulative KM estimate of remaining free of relapse was 91% (95% CI 83–95) and 81% (95% CI 72–87) in the HSCT and in the OCR groups, respectively. The E‐value RR for unmeasured confounding was 4.7 for the ARR ratio and 1.6 for its CI lower limit.

During the mean 3 years of follow up, the proportion of patients without new MRI activity was very high in both groups. After 3 years from therapy initiation, the weighted cumulative KM estimate of remaining free of new MRI activity was 93% (95% CI 88–98) versus 93% (95% CI 91–96) in the AHSCT and OCR groups, respectively (Fig 3C). Patients in the AHSCT and OCR groups had similar risk of experiencing new MRI activity (HR 1·03, 95% CI 0.32–3.25; *p* = 0.96).

The proportion of patients experiencing disability accumulation and disability improvement was also similar in the AHSCT and OCR groups. The KM analysis showed a similar weighted cumulative estimate of 6‐month confirmed EDSS worsening (at 3 years: 24%, 95% CI 17–35 vs. 19%, 95% CI 15–25; *p* = 0.86; Fig 3D) and of 6‐month EDSS improvement (at 3 years: 13%, 95% CI 7–23 vs. 13%, 95% CI 7–17; *p* = 0.77) (Fig 3E) in the AHSCT and OCR groups, respectively. At the last follow up, the mean EDSS change from baseline was 0.068 (SD 1.10) for AHSCT patients and 0.12 (SD 0.78) for those receiving OCR, whereas the median EDSS change was 0 (IQR 0–0.5) and 0 (IQR 0–0.5), respectively.

Finally, patients treated with AHSCT had a lower, but not statistically significant, weighted risk of NEDA‐3 status failure compared with the OCR group (HR 0.62, 95% CI 0.35–1.09; *p* = 0.095; Fig 2F). At 3 years from baseline, the estimated proportion of patients experiencing NEDA was 73% (95% CI 63–81) and 65% (95% CI 58–70) in the AHSCT and OCR groups, respectively.

### Sensitivity Analyses

Analyses were also performed on crude data before propensity score matching. The unweighted KM curves comparing AHSCT with ATZ (Fig S1A–D) and with OCR (Fig S2A–D) for all endpoints are presented in supplemental material; the estimates from both the unweighted and weighted samples were very similar.

In addition, as we were not able to use a multinomial logistic regression model for propensity matching (see Statistical Analysis), we performed sensitivity analyses after computing generalized boosted model‐based propensity scores (Table S4). Sensitivity analyses results were in agreement with the main results and confirmed superior efficacy of AHSCT compared with ATZ and to OCR (Table S5). Notably, sensitivity analyses also showed statistically significant greater effect of AHSCT versus OCR on time to new relapses (Table S5).

### Adverse Events after AHSCT

In the AHSCT group, 95% (n = 98) of patients experienced at least 1 adverse event (Table S6). Within 15 days from AHSCT conditioning, 5 patients required intensive care unit admission because of sepsis shock (n = 3) and pulmonary edema with respiratory failure (n = 2). Additionally, 78 patients experienced infections, particularly with Epstein–Barr virus reactivation, 71 patients had fluid overload, and 66 patients suffered from severe non‐infective diarrhea. Late adverse events included the development of secondary autoimmune diseases in 10 patients, including autoimmune thyroiditis (n = 6), Graves' disease (n = 1), and autoimmune thrombocytopenia (n = 3). In the study cohort, 1 patient (woman, age 51 years, EDSS 6.5) died 32 days after PBSC reinfusion and was not included in the analysis due to exclusion criteria (short follow up). AHSCT expectedly involved higher upfront risk during and shortly after this treatment procedure. Comparison of safety and tolerability of AHSCT with ATZ and OCR was not an aim of this study.

## Discussion

Case series, observational studies, and trials have demonstrated that AHSCT can effectively halt clinical and subclinical inflammatory disease activity in MS patients not responding to conventional DMTs.^28^ Its use has been recommended by the American Society for Blood and Marrow Transplantation as “standard of care,” even for untreated patients with negative prognostic features and at high risk of developing severe disability.^29^ Advances in patients selection, treatment procedures, and management of risks and adverse events have improved the transplant safety profile,^6^ imposing a reassessment of the AHSCT position in the MS treatments algorithm. In 2 previous trials, the ASTIMS and MIST, AHSCT was shown to be more effective in suppressing the disease inflammatory parameters compared with mitoxantrone,^16^ which is no longer used in MS management due to its toxicity, or compared with a heterogenous group of MS patients treated with a wide range of DMTs, among which only 54% received high‐efficacy medications.^17^ Overall, clear indications as to whether and when clinicians should favor AHSCT as an alternative to highly active DMTs remain unclear, and only clinical trials can definitively answer the question.

In the present retrospective real‐world study, we implemented PSOW, which is a robust method for controlling confounders in observational studies, ensuring homogeneity across treatment groups. We demonstrated, among patients with relapsing forms of MS, superior efficacy of AHSCT in preventing clinical relapses compared with 2 high‐efficacy DMTs, ATZ and OCR. During the 5‐year follow‐up period, patients who received AHSCT, compared with those who were treated with ATZ, had significantly lower mean ARR (0.020 vs 0.078), and in significantly larger proportions remained free of new relapses (89% vs 63%) and of new MRI activity (85% vs 71%). The difference between the 2 groups became mainly evident after the first 2 years from baseline, when in the ATZ group, following the completion of the second course of treatment, an increasingly larger number of patients started to experience new clinical and subclinical inflammatory activity. Similarly, during the shorter follow‐up period (3 mean years), in the AHSCT group, compared with the OCR group, we observed significantly lower mean ARR (0·026 vs 0·067), and, albeit not statistically significant, a larger proportion of patients free of relapses (91% vs 81%), suggesting a trend to reduced probability of acute attacks. Both the AHSCT and the OCR groups performed similarly in preventing new MRI activity. The robustness of the superior effect of AHSCT on ARR is confirmed by the high E‐value RR for unmeasured confounding in both the ATZ (7.2) and OCR (4.7) analyses, indicating that a potential unknown confounder, to nullify results, should have a strong association with both the treatment and the outcome, which is an unlikely scenario.

Reassuringly, sensitivity analyses of unweighted data (Figs. S1A–E and S2A–E) and of weighed data estimated from generalized boosted model‐based propensity scores (Tables S4 and S5) were in complete agreement with our main analysis results, indicating little bias affecting our data. Notably, these also showed a statistically significant greater effect of AHSCT compared with OCR, not only on the ARR, but also on the risk of clinical relapses (Table S5), which further confirms the superior efficacy of the transplant in preventing mechanisms determining the occurrence of inflammatory attacks.

In contrast to the analyses of inflammatory (clinical relapses and MRI activity) outcomes, AHSCT was not associated with better outcomes on disability accumulation. Confirmed EDSS worsening occurred in similar proportions in the AHSCT and in the 2 DMTs comparators groups, indicating that, among patients who received the transplant, the more profound relapse suppression compared with ATZ and OCR did not significantly impact on the accumulation of permanent disability. The therapeutic effect of AHSCT might be exclusively related to the prevention of mechanisms underlying the relapses‐associated worsening, with little influence on neurodegenerative processes accounting for the progression independent of relapsing activity, which gradually becomes more prominent late in the disease course.^30^, ^31^ However, due to referral practice and treatment selection criteria, most of the patients who received AHSCT had already failed responding to highly active treatments and, thus, had already accumulated severe disability (mean EDSS 5) at baseline. This implies that, although patients were still experiencing predominantly relapsing symptoms, they may have entered a subtle progressive course, albeit not fulfilling the formal definition of secondary progressive MS, and they were at high risk of disability progression unrelated to relapses, which is more likely to occur in the more advanced disease stage. Therefore, selection bias might have hindered any effect of AHSCT in preventing disability worsening. In earlier studies, younger age, shorter disease duration, and mild to moderate disability were shown to be features distinguishing patient groups more likely to benefit from AHSCT.^6^ Whether the transplant compared with highly effective DMTs can more effectively prevent the long‐term disability accumulation when administered early in the disease course and at younger age remains an open question. Presently, randomized controlled trials (RCTs) are ongoing (BEAT‐MS, NCT04047628;STAR‐MS, EudraCT: 2019‐001549‐42; RAM‐MS NCT03477500) comparing AHSCT with ATZ, OCR, natalizumab, cladribine, and rituximab, which will provide higher‐grade RCT evidence.

Interestingly, we observed that the use of AHSCT was associated with a significantly higher probability of experiencing EDSS improvement when compared with ATZ, although such difference was not observed in the comparison with OCR. This is in line with previous studies showing that AHSCT‐treated patients are more likely to experience disability improvement when compared with other DMTs, including ATZ.^32^, ^33^, ^34^ Mechanisms underlying tissue repair remain largely unclear, but it reasonable to assume that the suppression of chronic inflammatory activity, which is induced by central nervous system penetrant chemotherapy used during the AHSCT conditioning regime, has the potential to promote remyelination and neural plasticity.

The superior efficacy of AHSCT compared with ATZ is consistent with results from several previous studies analyzing smaller cohorts with shorter follow up.^32^, ^33^, ^34^ In contrast, a recent analysis from the MSBase provided comparative evidence of AHSCT versus OCR, showing similar efficacy of the 2 compounds in preventing new relapses and disability worsening.^18^ However, in this study, a large proportion of OCR‐treated patients (72%) dropped out of the analysis after 2 years from baseline because of short follow up. In addition, the lack of information on pre‐baseline therapy in a large number of patients (58.2% of AHSCT, and 57.5% of OCR cohorts), the considerable heterogeneity of AHSCT protocol (67% of the transplanted patients received intermediate‐intensity conditioning regimens, and in 16% of the patients, ATG was not used), and the lack of MRI data among propensity score matching criteria, and as an endpoint, represent important limitations, which might explain differences compared with the present study results.

We acknowledge that, in our analysis, the AHSCT group's observed superiority in suppressing inflammatory disease activity, compared with OCR, was less prominent than in comparison with ATZ. The ARR was significantly lower among those who had AHSCT compared with OCR, but the difference between the 2 groups in the cumulative proportion of patients free of relapses (91.3% vs 80.7%) did not reach statistical significance, although in sensitivity analyses, the transplant was shown to exert a statistically significant greater effect also on the time to new relapses (Table S5). In addition, when assessing the MRI outcomes, we observed a “flooring effect,” with only 6.7% of patients experiencing MRI activity in both the AHSCT and OCR groups. This is consistent with a strong efficacy of both treatments on MRI lesions development, and it is further explained by the relatively short follow‐up period in the OCR cohort, as most of the patients in our analysis were censored 3 years after therapy initiation. With extended follow up, it is reasonable to expect that the AHSCT superiority, compared with OCR, in preventing focal inflammatory activity could emerge more clearly, but further confirmatory studies with longer observation period are needed.

Different pharmacokinetic properties must also be taken into consideration, as AHSCT is a one‐off procedure able to induce immune changes sustained over time, while OCR requires regular treatment administrations to exert its therapeutic effect. Although formal cost‐effectiveness analyses are lacking, the cost of a one‐off therapy with comparable effectiveness may be lower than long‐term treatment with B‐cell depleting licensed agents.^35^

In addition, over the past years there has been a substantial improvement of AHSCT‐related mortality and management of adverse events.^4^ Among our AHSCT‐treated patients, the incidence of immediate and late adverse events was not in excess of the rates reported in literature, including the observed (1) death, indicating a treatment‐related mortality of 1.1%, which is within the range of previous reports. Indeed, in a recent meta‐analysis including 4,831 patients from 50 studies, the pooled treatment‐related mortality was 4.0% (95% CI 2–6%), a number influenced by the high mortality reported in earlier studies.^36^ In contrast, safety concerns have been raised related to the long‐term depletion of B cells, and consequent hypogammaglobulinemia and neutropenia, with associated risk of impaired response to vaccines,^37^ and of serious infections and neoplasms, especially among older patients,^38^, ^39^ which could be potentially mitigated by improving the selection of patients and by adopting an extended dosing schedule.^40^ However, factors accounting for B‐cell suppression related complications remain largely unknown, hindering the possibility of propensity matching patients based on the risk of side effects.

Arguably, the different degree of effectiveness of AHSCT compared with ATZ and OCR might be explained by the markedly different mechanisms of action of the 3 compounds. Patients undergoing AHSCT following the lympho/myeloablation experience an immune reset through the generation of new self‐tolerant T and B precursors from the reinfused self‐hematopoietic stem cells,^6^ whereas after administration of ATZ, complete T‐cell recovery tends to occur within 3–4 years, and B‐cell recovery within the first year, without renewing the adaptive immune compartment.^41^ The OCR depletes B cells and 20% of circulating T cells expressing CD20 within 2 weeks from the first course,^42^ and data on immune reconstitution after rituximab suspension showed that the restored B‐cell compartment is mainly composed of naïve B cells, the levels of pro‐inflammatory cytokine is decreased, whereas anti‐inflammatory cytokines are increased.^43^, ^44^ The effect of AHSCT on the B‐cell compartment is largely unknown, and further studies are required to understand if its mechanism of action could, wholly or in part, depend on depletion and reconstitution of the B‐cell compartment.

Notably, AHSCT induced sustained disease stability in the majority of our cohort's patients, with 63.1% maintaining NEDA‐3 status and 93.3% not requiring further treatment within 5 years from the transplant (Table S1). Among patients who experienced disease activity after AHSCT, 7 subsequently received other DMTs, including natalizumab (n = 3), cyclophosphamide (n = 2), ocrelizumab (n = 1), and a second AHSCT (n = 1). In addition, 5 years after AHSCT, 85% of patients were still free of inflammatory disease activity (Table S1). Although it is reasonable to expect a few more “non‐responders” emerging in the longer term, it is unlikely that this will significantly impact the overall cost–benefit of the transplant.

In our cohort, all but 1 patient received cyclo‐ATG conditioning and 1 (the first patient) BEAM‐ATG. Previous European Bone Marrow Transplantation Society studies reported no significant differences in the efficacy or safety of cyclo‐ATG versus BEAM‐ATG used for autografting in MS.^15^, ^45^ Although different conditioning intensities may reveal differential efficacy or safety in other study settings (e.g. RCTs), the current evidence does not support major differences to be expected between lymphoablative (cyclo‐ATG) versus more intensive, partly myeloablative conditioning (BEAM‐ATG). It is possible that high‐intensity, fully myeloablative regimes, such as busulfan–cyclophosphamide, could be more effective, but their higher risk profile has so far limited their utilization to 1 experienced center in Canada only.^46^

The retrospective design, lack of true randomization, and varied follow‐up durations among treatment groups may restrict the generalizability of our results. We have attempted to reduce treatment–indication bias by including the most relevant variables in the PSOW calculation, but we cannot entirely rule out the possibility that other unmeasured prognostic factors and sources of bias may have affected the results. In addition, although PSOW is considered a robust method for controlling confounders in observational studies, we acknowledge the limitation of our statistical approach, which implied analyzing modelled data rather than directly comparing the crude real‐world data. However, results from analyses of unweighted crude data (Figs. S1A–E and S2A–E) were reassuringly in agreement with our main analyses of weighted data. Although some analyses may have had low statistical power mainly due to the relatively small sample size of the AHSCT group compared with the majority of previously published studies, we are here reporting data from 1 of the largest cohorts of AHSCT treated patients.^11^, ^14^, ^15^, ^16^, ^18^, ^32^ In addition, unlike many other studies, in our cohort, AHSCT was prescribed to patients who fulfilled predefined inclusion criteria, which were evaluated by a multidisciplinary team panel for each single case, ensuring homogeneity of access to the transplant. Further strengths of our study include the uniform AHSCT protocol administered at 2 European Bone Marrow Transplantation Society accredited centers, the inclusion of MRI data at baseline and on disease monitoring, the clinical assessments performed by 5 experienced MS physicians at a single MS tertiary center, covering a well ascertained geographic area (southwest London), which reduces the possibility of ascertainment bias and of interrater variability of EDSS scores. Importantly, in our analysis of non‐responders (Tables S2 and S3), a larger proportion in the AHSCT group compared with the ATZ and OCR groups previously had disease activity while being on highly active DMTs (66.7%, 45.7%, and 41.3%, respectively), indicating a more aggressive disease course and, therefore, higher risk of experiencing further clinical and radiological breakthrough. Overall, this might have downsized the AHSCT suppressive effect on relapses and MRI activity.

The results allow us to draw conclusions separately about the effectiveness of AHSCT against 2 high‐efficacy DMTs in a typical clinical scenario of patients experiencing the relapsing form of MS with highly active disease previously not adequately controlled by treatments. Our data showing that the superior efficacy of AHSCT suggested by a number of indirect comparisons is not clear‐cut, and corroborates the rationale for RCTs to confirm their equipoise. The RCTs are expected to provide the definitive, high‐quality evidence to support clinical decision‐making for treatment of individuals with highly active MS.

## Author Contributions

P.A.M., A.Z., R.N., and A.Sc. contributed to the conception and design of the study. All authors contributed to acquisition and analysis of data. P.A.M., A.Z., A.Si., R.N., and A.Sc. contributed to drafting the text or preparing the figures.

## Potential Conflicts of Interest

Nothing to report.

## Supporting information


**DATA S1** Supporting Information.

## Data Availability

Data collected for the study will be made available upon reasonable request to the corresponding author.

## References

[1] Jakimovski D , Bittner S , Zivadinov R , et al. Multiple sclerosis. Lancet 2024;403:183–202. 10.1016/S0140-6736(23)01473-3. 37949093

[2] Rotstein DL , Healy BC , Malik MT , et al. Evaluation of no evidence of disease activity in a 7‐year longitudinal multiple sclerosis cohort. JAMA Neurol 2015;72:152–158. 10.1001/jamaneurol.2014.3537. 25531931

[3] Cencioni MT , Genchi A , Brittain G , et al. Immune reconstitution following autologous hematopoietic stem cell transplantation for multiple sclerosis: a review on behalf of the EBMT autoimmune diseases working party. Front Immunol 2022;12:813957. 10.3389/fimmu.2021.813957. 35178046 PMC8846289

[4] Sormani MP , Muraro PA , Schiavetti I , et al. Autologous hematopoietic stem cell transplantation in multiple sclerosis. Neurology 2017;88:2115–2122. 10.1212/WNL.0000000000003987. 28455383

[5] Sharrack B , Saccardi R , Alexander T , et al. Autologous haematopoietic stem cell transplantation and other cellular therapy in multiple sclerosis and immune‐mediated neurological diseases: updated guidelines and recommendations from the EBMT Autoimmune Diseases Working Party (ADWP) and the Joint Accreditation Committee of EBMT and ISCT (JACIE). Bone Marrow Transplant 2020;55:283–306. 10.1038/s41409-019-0684-0. 31558790 PMC6995781

[6] Muraro PA , Martin R , Mancardi GL , et al. Autologous haematopoietic stem cell transplantation for treatment of multiple sclerosis. Nat Rev Neurol 2017;13:391–405. 10.1038/nrneurol.2017.81. 28621766

[7] Moore JJ , Massey JC , Ford CD , et al. Prospective phase II clinical trial of autologous haematopoietic stem cell transplant for treatment refractory multiple sclerosis. J Neurol Neurosurg Psychiatry 2019;90:514–521. 10.1136/jnnp-2018-319446. 30538138

[8] Tolf A , Fagius J , Carlson K , et al. Sustained remission in multiple sclerosis after hematopoietic stem cell transplantation. Acta Neurol Scand 2019;140:320–327. 10.1111/ane.13147. 31297793

[9] Kvistad SAS , Lehmann AK , Trovik LH , et al. Safety and efficacy of autologous hematopoietic stem cell transplantation for multiple sclerosis in Norway. Mult Scler J 2020;26:1889–1897. 10.1177/1352458519893926. 31833798

[10] Das J , Snowden JA , Burman J , et al. Autologous haematopoietic stem cell transplantation as a first‐line disease‐modifying therapy in patients with ‘aggressive’ multiple sclerosis. Mult Scler J 2021;27:1198–1204. 10.1177/1352458520985238. PMC8226372 33565902

[11] Burt RK , Han X , Quigley K , et al. Real‐world application of autologous hematopoietic stem cell transplantation in 507 patients with multiple sclerosis. J Neurol 2022;269:2513–2526. 10.1007/s00415-021-10820-2. 34633525 PMC8503710

[12] Silfverberg T , Zjukovskaja C , Ljungman P , et al. Haematopoietic stem cell transplantation for treatment of relapsing‐remitting multiple sclerosis in Sweden: an observational cohort study. J Neurol Neurosurg Psychiatry 2024;95:125–133. 10.1136/jnnp-2023-331864. 37748927 PMC10850659

[13] Kvistad CE , Lehmann AK , Kvistad SAS , et al. Autologous hematopoietic stem cell transplantation for multiple sclerosis: long‐term follow‐up data from Norway. Mult Scler J 2024;30:751–754. 10.1177/13524585241231665. PMC11071593 38345003

[14] Boffa G , Massacesi L , Inglese M , et al. Long‐term clinical outcomes of hematopoietic stem cell transplantation in multiple sclerosis. Neurology 2021;96:e1215–e1226. 10.1212/WNL.0000000000011461. 33472915

[15] Silfverberg T , Zjukovskaja C , Noui Y , et al. BEAM or cyclophosphamide in autologous haematopoietic stem cell transplantation for relapsing‐remitting multiple sclerosis. Bone Marrow Transplant 2024;59:125–133. 10.1038/s41409-024-02397-x. 39187603 PMC11530369

[16] Mancardi GL , Sormani MP , Gualandi F , et al. Autologous hematopoietic stem cell transplantation in multiple sclerosis. Neurology 2015;84:981–988. 10.1212/WNL.0000000000001329. 25672923

[17] Burt RK , Balabanov R , Burman J , et al. Effect of nonmyeloablative hematopoietic stem cell transplantation vs continued disease‐modifying therapy on disease progression in patients with relapsing‐remitting multiple sclerosis: a randomized clinical trial. JAMA 2019;321:165–174. 10.1001/jama.2018.18743. 30644983 PMC6439765

[18] Kalincik T , Sharmin S , Roos I , et al. Comparative effectiveness of autologous hematopoietic stem cell transplant vs Fingolimod, Natalizumab, and Ocrelizumab in highly active relapsing‐remitting multiple sclerosis. JAMA Neurol 2023;80:702–713. 10.1001/jamaneurol.2023.1184. 37437240 PMC10186210

[19] Nicholas RS , Rhone EE , Mariottini A , et al. Autologous hematopoietic stem cell transplantation in active multiple sclerosis. Neurology 2021;97:e890–e901. 10.1212/WNL.0000000000012449. 34253634 PMC8408506

[20] Thomas LE , Li F , Pencina MJ . Overlap weighting: a propensity score method that mimics attributes of a randomized clinical trial. JAMA 2020;323:2417–2418. 10.1001/jama.2020.7819. 32369102

[21] Coles AJ , Cohen JA , Fox EJ , et al. Alemtuzumab CARE‐MS II 5‐year follow‐up. Neurology 2017;89:1117–1126. 10.1212/WNL.0000000000004354. 28835403 PMC5595276

[22] Hauser SL , Kappos L , Arnold DL , et al. Five years of ocrelizumab in relapsing multiple sclerosis. Neurology 2020;95:e1854–e1867. 10.1212/WNL.0000000000010376. 32690791 PMC7682822

[23] Thompson AJ , Banwell BL , Barkhof F , et al. Diagnosis of multiple sclerosis: 2017 revisions of the McDonald criteria. Lancet Neurol 2018;17:162–173. 10.1016/S1474-4422(17)30470-2. 29275977

[24] Kurtzke JF . Rating neurologic impairment in multiple sclerosis: an expanded disability status scale (EDSS). Neurology 1983;33:1444. 10.1212/WNL.33.11.1444. 6685237

[25] McCaffrey DF , Griffin BA , Almirall D , et al. A tutorial on propensity score estimation for multiple treatments using generalized boosted models. Stat Med 2013;32:3388–3414. 10.1002/sim.5753. 23508673 PMC3710547

[26] Giovannoni G , Lang S , Wolff R , et al. A systematic review and mixed treatment comparison of pharmaceutical interventions for multiple sclerosis. Neurol Ther 2020;9:359–374. 10.1007/s40120-020-00212-5. 32989721 PMC7606402

[27] Vander Weele TJ , Ding P . Sensitivity analysis in observational research: introducing the E‐value. Ann Intern Med 2017;167:268–274. 10.7326/M16-2607. 28693043

[28] Cohen JA , Cross AH . Is autologous hematopoietic stem cell transplant better than high‐efficacy disease‐modifying therapies for relapsing multiple sclerosis? JAMA Neurol 2023;80:669–672. 10.1001/jamaneurol.2023.0467. 37184849

[29] Cohen JA , Baldassari LE , Atkins HL , et al. Autologous hematopoietic cell transplantation for treatment‐refractory relapsing multiple sclerosis: position statement from the American Society for Blood and Marrow Transplantation. Biol Blood Marrow Transplant 2019;25:845–854. 10.1016/j.bbmt.2019.02.014. 30794930

[30] Tur C , Carbonell‐Mirabent P , Cobo‐Calvo Á , et al. Association of early progression independent of relapse activity with long‐term disability after a first demyelinating event in multiple sclerosis. JAMA Neurol 2023;80:151–160. 10.1001/jamaneurol.2022.4655. 36534392 PMC9856884

[31] Portaccio E , Magyari M , Havrdova EK , et al. Multiple sclerosis: emerging epidemiological trends and redefining the clinical course. Lancet Reg Health Eur 2024;44:100977. 10.1016/j.lanepe.2024.100977. 39444703 PMC11496978

[32] Boffa G , Signori A , Massacesi L , et al. Hematopoietic stem cell transplantation in people with active secondary progressive multiple sclerosis. Neurology 2023;100:e1109–e1122. 10.1212/WNL.0000000000206750. 36543569 PMC10074454

[33] Häußler V , Ufer F , Pöttgen J , et al. aHSCT is superior to alemtuzumab in maintaining NEDA and improving cognition in multiple sclerosis. Ann Clin Transl Neurol 2021;8:1269–1278. 10.1002/acn3.51366. 33949790 PMC8164852

[34] Zhukovsky C , Sandgren S , Silfverberg T , et al. Autologous haematopoietic stem cell transplantation compared with alemtuzumab for relapsing–remitting multiple sclerosis: an observational study. J Neurol Neurosurg Psychiatry 2021;92:189–194. 10.1136/jnnp-2020-323992. 33106366 PMC7841472

[35] Burt RK , Tappenden P , Han X , et al. Health economics and patient outcomes of hematopoietic stem cell transplantation versus disease‐modifying therapies for relapsing remitting multiple sclerosis in the United States of America. Mult Scler Relat Disord 2020;45:102404. 10.1016/j.msard.2020.102404. 32731201

[36] Nabizadeh F , Pirahesh K , Rafiei N , et al. Autologous hematopoietic stem‐cell transplantation in multiple sclerosis: a systematic review and meta‐analysis. Neurol Ther 2022;11:1553–1569. 10.1007/s40120-022-00389-x. 35902484 PMC9333355

[37] Bar‐Or A , Calkwood JC , Chognot C , et al. Effect of ocrelizumab on vaccine responses in patients with multiple sclerosis. Neurology 2020;95:e1999–e2008. 10.1212/WNL.0000000000010380. 32727835 PMC7843152

[38] Prosperini L , Haggiag S , Tortorella C , et al. Age‐related adverse events of disease‐modifying treatments for multiple sclerosis: a meta‐regression. Mult Scler J 2021;27:1391–1402. 10.1177/1352458520964778. 33104449

[39] Alvarez E , Longbrake EE , Rammohan KW , et al. Secondary hypogammaglobulinemia in patients with multiple sclerosis on anti‐CD20 therapy: pathogenesis, risk of infection, and disease management. Mult Scler Relat Disord 2023;79:105009. 10.1016/j.msard.2023.105009. 37783194

[40] Lycke J , Svenningsson A . Long‐term treatment with anti‐CD20 monoclonal antibodies is untenable because of risk: commentary. Mult Scler J 2022;28:1177–1178. 10.1177/13524585221101138. PMC9189590 35678609

[41] Thompson SAJ , Jones JL , Cox AL , et al. B‐cell reconstitution and BAFF after Alemtuzumab (Campath‐1H) treatment of multiple sclerosis. J Clin Immunol 2010;30:99–105. 10.1007/s10875-009-9327-3. 19763798

[42] Gingele S , Jacobus TL , Konen FF , et al. Ocrelizumab depletes CD20+ T cells in multiple sclerosis patients. Cells 2019;8:12. 10.3390/cells8010012. PMC6356421 30597851

[43] Bar‐Or A , Fawaz L , Fan B , et al. Abnormal B‐cell cytokine responses a trigger of T‐cell–mediated disease in MS? Ann Neurol 2010;67:452–461. 10.1002/ana.21939. 20437580

[44] Li R , Rezk A , Miyazaki Y , et al. Proinflammatory GM‐CSF–producing B cells in multiple sclerosis and B cell depletion therapy. Sci Transl Med 2015;7:310ra166. 10.1126/scitranslmed.aab4176. 26491076

[45] Saccardi R , Badoglio M , Burman J , et al. BEAM vs cyclophosphamide‐based conditioning regimen in aggressive multiple sclerosis: a retrospective analysis of European blood and marrow transplantation society. Blood 2019;134:3313. 10.1182/blood-2019-125233.

[46] Atkins HL , Bowman M , Allan D , et al. Immunoablation and autologous haemopoietic stem‐cell transplantation for aggressive multiple sclerosis: a multicentre single‐group phase 2 trial. Lancet 2016;388:576–585. 10.1016/S0140-6736(16)30169-6. 27291994

