# Supplementary material for: Effectiveness of Autologous Hematopoietic Stem Cell Transplantation versus Alemtuzumab and Ocrelizumab in Relapsing Multiple Sclerosis: A Single Center Cohort Study
Source: Ann Neurol. 2025 Apr 19;98(2):294–307. doi: 10.1002/ana.27247 (PMC12278282; doi:10.1002/ana.27247)

**SUPPLEMENTARY INFORMATION**

**Index of Contents:**

**Table S1:** Summary of three cohorts’ outcomes during follow-up period before PSOW analysis

**Table S2:** Clinical and radiological features in patients with NEDA 2 failure

**Table S3:** Clinical and radiological features in patients with EDSS progression

**Table S4:** Weighted characteristics of the three treatment groups after Generalized Boosted Models

**Table S5:** Sensitivity analyses using weights estimated from Generalized Boosted Model-based propensity scores

**Table S6:** Adverse events among patients who received AHSCT

**Figure S1A**: unweighted KM analysis AHSCT vs ATZ: time to relapse

**Figure S1B**: unweighted KM analysis AHSCT vs ATZ: time to new MRI activity

**Figure S1C**: unweighted KM analysis AHSCT vs ATZ: time to EDSS progression

**Figure S1D**: unweighted KM analysis AHSCT vs ATZ: time to EDSS improvement

**Figure S1E**: unweighted KM analysis AHSCT vs ATZ: time to NEDA failure

**Figure S2A**: unweighted KM analysis AHSCT vs OCR: time to relapse

**Figure S2B**: unweighted KM analysis AHSCT vs OCR: time to new MRI activity

**Figure S2C**: unweighted KM analysis AHSCT vs OCR: time to EDSS progression

**Figure S2D**: unweighted KM analysis AHSCT vs OCR: time to EDSS improvement

**Figure S2E**: unweighted KM analysis AHSCT vs OCR: time to NEDA failure

**Table S1. Summary of clinical and radiological outcomes during follow-up period of the three cohorts before PSOW analysis**

| Variables | AHSCT | ATZ | OCR |
| --- | --- | --- | --- |
| ARR mean (CI 95%) | 0·030 (0·012-0·048) | 0·075 (0·055-0·095) | 0·071 (0·055-0·088) |
| New relapse n (%) | 10 (9·7) | 52 (25·5) | 56 (17·8) |
| New MRI activity n (%) | 10 (9·7) | 56 (27) | 21 (4·8) |
| EDSS Progression n (%) | 32 (31·1) | 59 (28·9%) | 75 (17·1) |
| NEDA failure n (%) | 38 (36·9) | 106 (52·0%) | 125 (28·5) |
| Switch Therapy n (%) | 7 (6·7) | 59 (29·3) | 16 (5·1) |

**Table S2. Clinical and radiological features of patients with NEDA 2 failure (new relapses and MRI activity)**

| Variables at baseline | HSCT | ATZ | OCR |
| --- | --- | --- | --- |
| N. | 15 | 70 | 63 |
| Sex, F/M (%) | 53·3/46·7 | 70/30 | 71·4/28·6 |
| Age, mean years (SD) | 40·6 (12) | 38·8 (8·7) | 46·3 (12·1) |
| MS duration, mean years (SD) | 11 (5·4) | 9·2 (6·7) | 14·1(9·0) |
| EDSS, median (25th-75th percentiles) | 6 (3-6) | 2·5(1·5-6) | 3·5(2-6) |
| EDSS change from previous 2 years, mean (SD) | 0·10 (0·5) | 0·40 (0·95) | 0·1 (0·8) |
| MRI activity during 2 years before treatment | 86·7 % | 87·1 % | 69·8 |
| 1 new T2 lesion | 33·3% | 44·3 % | 17·5 |
| ≥2 new T2 lesion | 53·3 % | 42·9 % | 12·7 |
| ARR during 2 years before treatment, mean (SD) | 0·55 (0·45) | 0·65 (0·36) | 0·5 (0·39) |
| Treatment Naïve | 20·0 % | 22·9 % | 7·9 |
| Number of previous DMTs, mean (SD), median | 2 (1·6), 2 | 1·09 (0·75), 1 | 1·5 (0·64) |
| High efficacy DMTs | 66·7% | 45·7% | 41·3% |

SD: standard deviation

**Table S3. Clinical and radiological features in patients with EDSS progression**

| Variables at baseline | HSCT | ATZ | OCR |
| --- | --- | --- | --- |
| N. | 37 | 68 | 62 |
| Sex, F/M (%) | 59·5/40·5 | 64·7/35·3 | 58·1/41·9 |
| Age, mean years (SD) | 41·6 (9·5) | 43·3 (9·6) | 45·6 (11·6) |
| MS duration, mean years (SD) | 10·8 (6·4) | 9·8 (6·4) | 11·4 (7·9) |
| EDSS, median (25th-75th percentiles) | 6 (3·5-6·5) | 3(2-5·5) | 3 (2-6) |
| EDSS change from previous 2 years, mean (SD) | 0·10 (0·78) | 0·30 (0·75) | 0·20 (0·79) |
| MRI activity during 2 years before treatment | 78·4 % | 58·8 % | 41·9 |
| 1 new T2 lesion | 33·8% | 25·0% | 22·6 |
| ≥2 new T2 lesion | 48·6 % | 29·7 % | 19·4 |
| ARR during 2 years before treatment, mean (SD) | 0·48 (0·48) | 0·50 (0·37) | 0·47 (0·38) |
| Treatment Naïve | 20·0 % | 22·9 % | 19·4 |
| Number of previous DMTs, mean (SD), median | 2·14 (1·29), 2 | 1·24 (0·67), 1 | 1·23 (0·75),1 |
| High efficacy DMTs | 67·6% | 41·2% | 25·8 |

SD: standard deviation

**Table S4. Weighted characteristics of the three treatment groups after Generalized Boosted Models**

**Table S5. Sensitivity analyses using weights estimated from Generalized Boosted Model-based propensity scores**

| **Endpoint** | **ARR (95% CI) / IRR (95% CI)** |
| --- | --- |
| **Annualized Relapse Rate** |  |
| HSCT | 0.015 (0.000-0.033) |
| ATZ | 0.068 (0.049-0.088) |
| OCR | 0.076 (0.055-0.097) |
| HSCT vs ATZ | 0.22 (0.06-0.78); p=0.020 |
| HSCT vs OCR | 0.19 (0.05-0.70); p=0.012 |
|  | **HR (95% CI); p-value** |
| **Time to first relapse** |  |
| HSCT vs ATZ | 0.21 (0.06-0.82); p=0.024 |
| HSCT vs OCR | 0.22 (0.06-0.84); p=0.027 |
|  |  |
| **Time to MRI activity** |  |
| HSCT vs ATZ | 0.42 (0.14-0.69); p=0.006 |
| HSCT vs OCR | 1.40 (0.42-4.64); p=0.58 |
|  |  |
| **Time to EDSS progression** |  |
| HSCT vs ATZ | 0.87 (0.44-1.71); p=0.69 |
| HSCT vs OCR | 1.08 (0.54-2.16); p=0.83 |
|  |  |
| **Time to NEDA** |  |
| HSCT vs ATZ | 0.50 (0.27-0.96); p=0.037 |
| HSCT vs OCR | 0.61 (0.32-1.17); p=0.14 |
|  |  |
| **Time to improvement** |  |
| HSCT vs ATZ | 4.89 (1.66-14.49); p=0.004 |
| HSCT vs OCR | 1.10 (0.43-2.81); p=0.84 |

**Table S6. Adverse events among patients who received ASCHT**

| **Adverse events** | **Number of events (%)** |
| --- | --- |
| **Infective AE** | |
| EBV reactivation n (%) | 78 (75·7) |
| Fever with positive blood culture n (%) | 43 (41·7) |
| Urinary tract infection n (%) | 22 (21·3) |
| Pneumonia n (%) | 15 (14·5) |
| CMV reactivation n (%) | 10 (10·3) |
| Skin bacterial infection n (%) | 10 (10·3) |
| Shingles n (%) | 9 (8·7) |
| Fungal infection n (%) | 6 (5·8) |
| Upper respiratory infection n (%) | 5 (4·8) |
| Influenza virus n (%) | 5 (4·8) |
| Clostridium Difficile n (%) | 5 (4·8) |
| Septic shock n (%) | 3 (2·9) |
| Herpes simplex n (%) | 2 (1·9) |
| **Gastrointestinal** | |
| Severe non infective diarrhea n (%) | 66 (64) |
| Nausea / vomiting n (%) | 25 (24·2) |
| Liver toxicity n (%) | 17 (16·5) |
| **Hematological** | |
| Venous thrombosis n (%) | 4 (3·8) |
| Thrombocytopenia autoimmune n (%) | 3 (2·9) |
| **Cardiovascular** | |
| Fluid overload n (%) | 71 (68·9) |
| Supraventricular tachycardia n (%) | 2 (1·9) |
| Atrial fibrillation n (%) | 1 (0·9) |
| **Endocrinological** | |
| Autoimmune Hypotiroidism n (%) | 6 (5·8) |
| Graves disease n (%) | 1 (0·9) |
| **Renal and urinary tract** | |
| Hematuria n (%) | 9 (8·7) |
| Acute renal insufficiency n (%) | 2 (1·9) |

**Figure S1A. Comparative analysis between AHSCT and ATZ; unweighted (from crude data) Kaplan Meier analysis. Cumulative probability of relapse.**


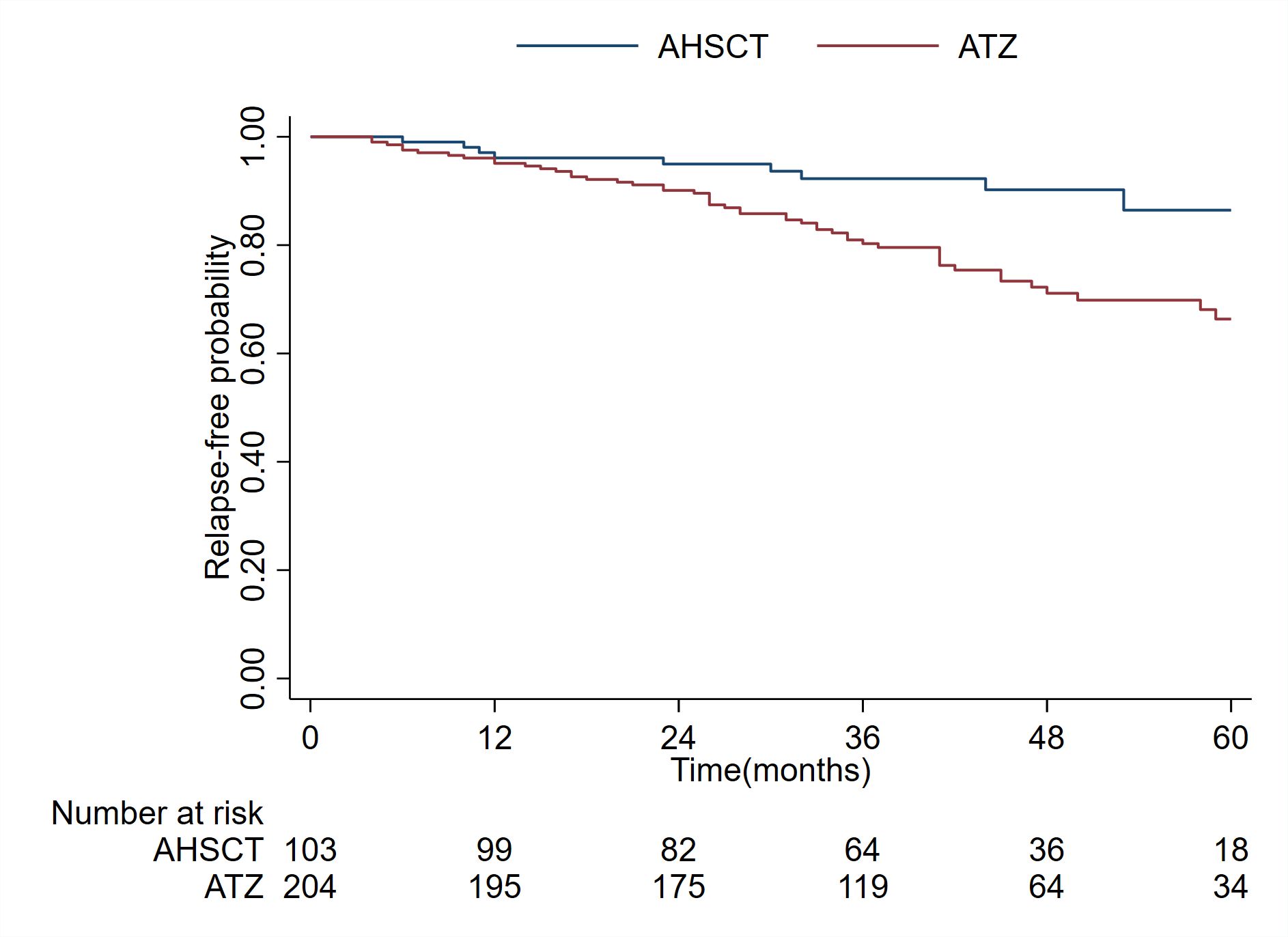


**Figure S1B. Comparative analysis between AHSCT and ATZ; unweighted (from crude data) Kaplan Meier analysis. Cumulative probability of new MRI activity**


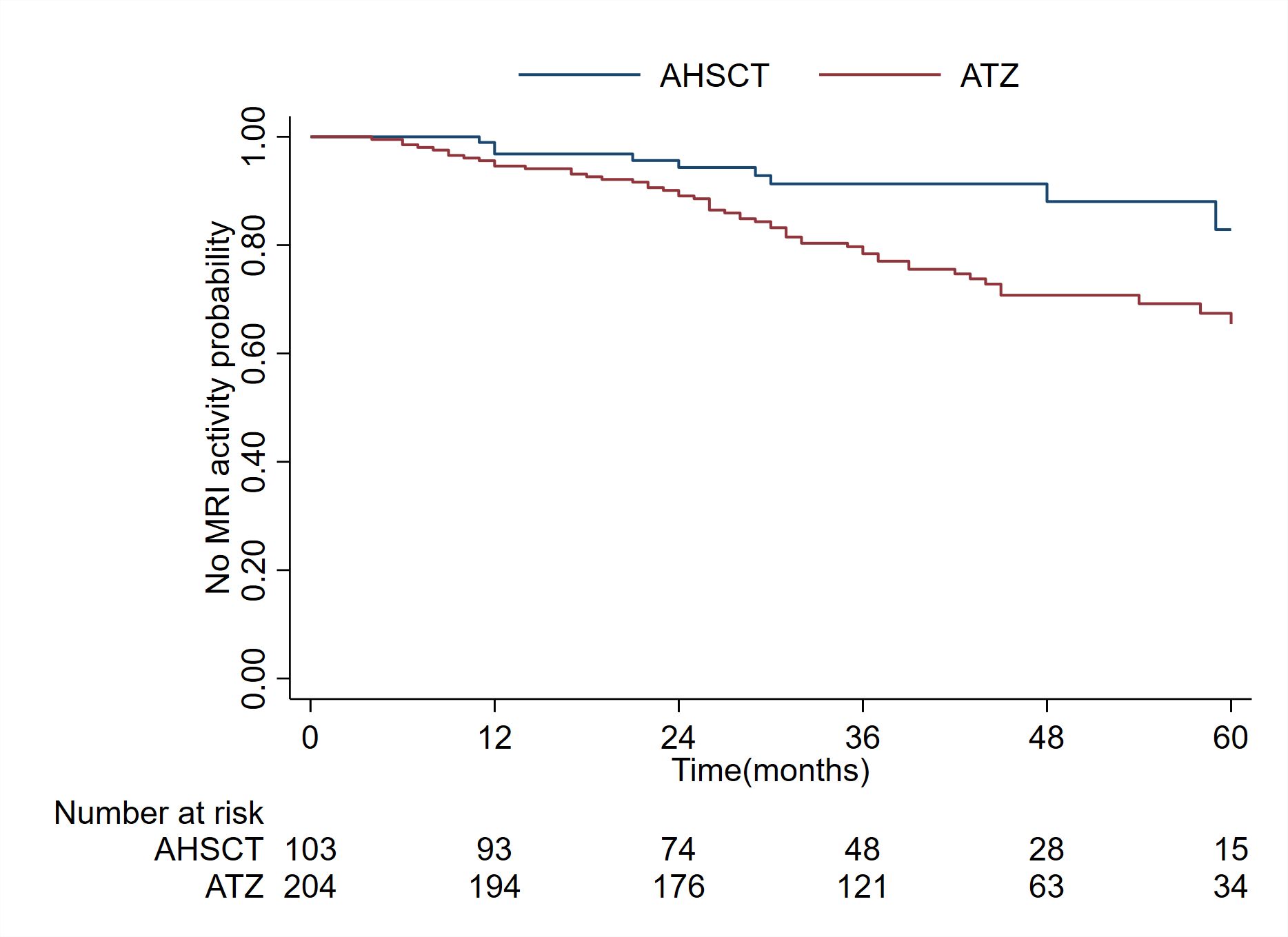


**Figure S1C. Comparative analysis between AHSCT and ATZ; unweighted (from crude data) Kaplan Meier analysis. Cumulative probability of EDSS progression**

**
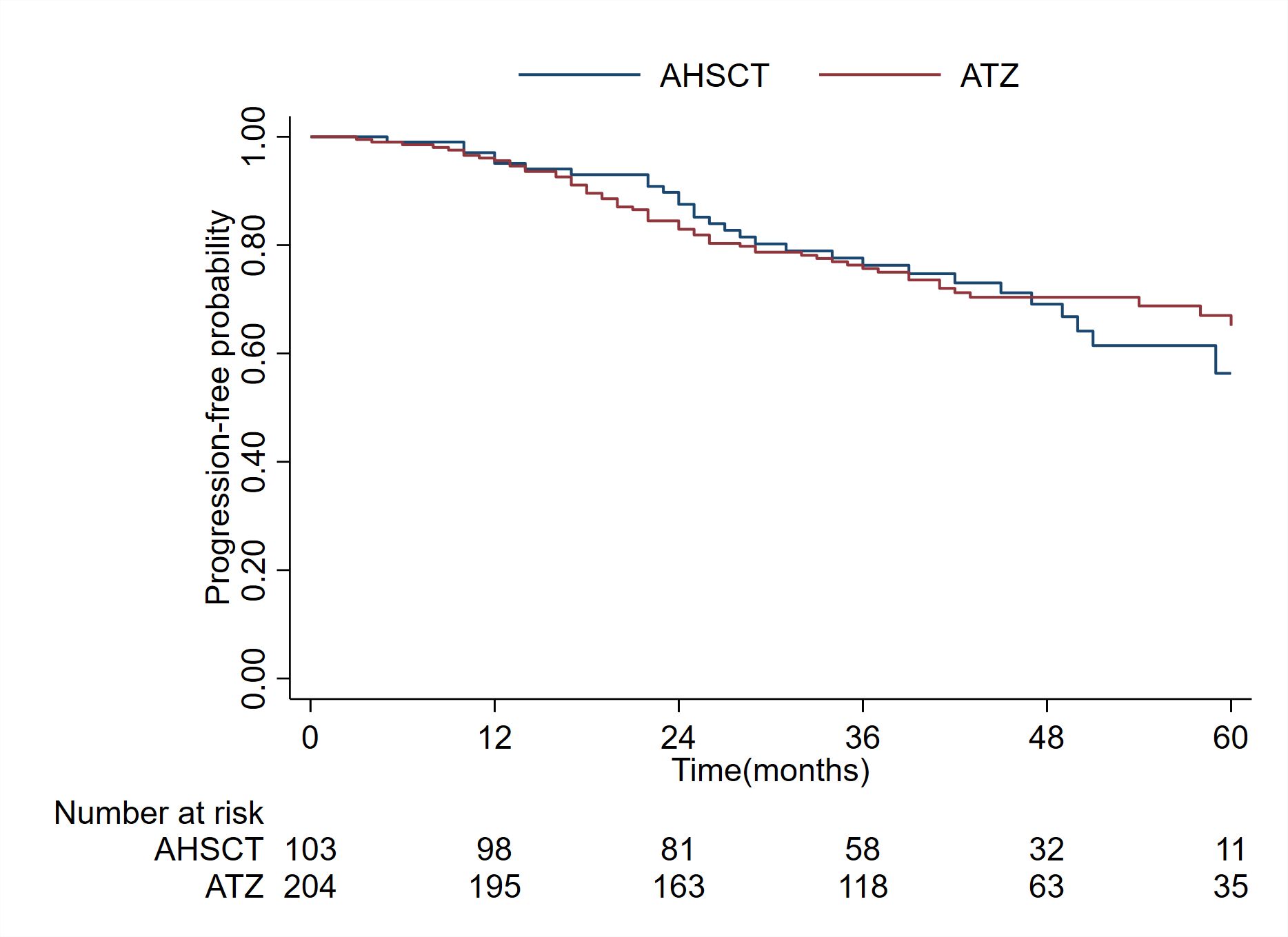
**

**Figure S1D. Comparative analysis between AHSCT and ATZ; unweighted (from crude data) Kaplan Meier analysis. Cumulative probability of EDSS improvement.**

**
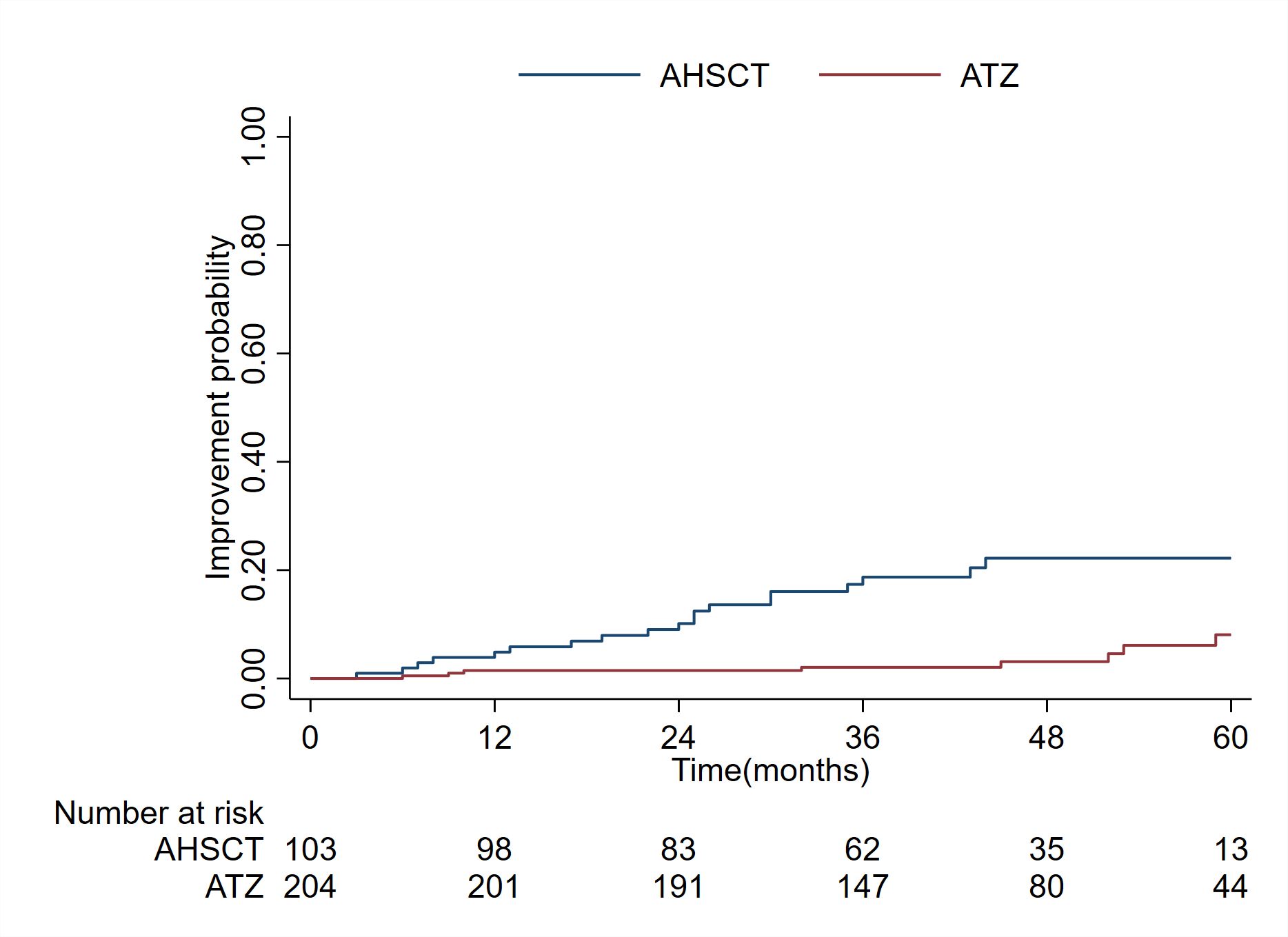
**

**Figure S1E. Comparative analysis between AHSCT and ATZ; unweighted (from crude data) Kaplan Meier analysis. Cumulative probability of NEDA failure.**


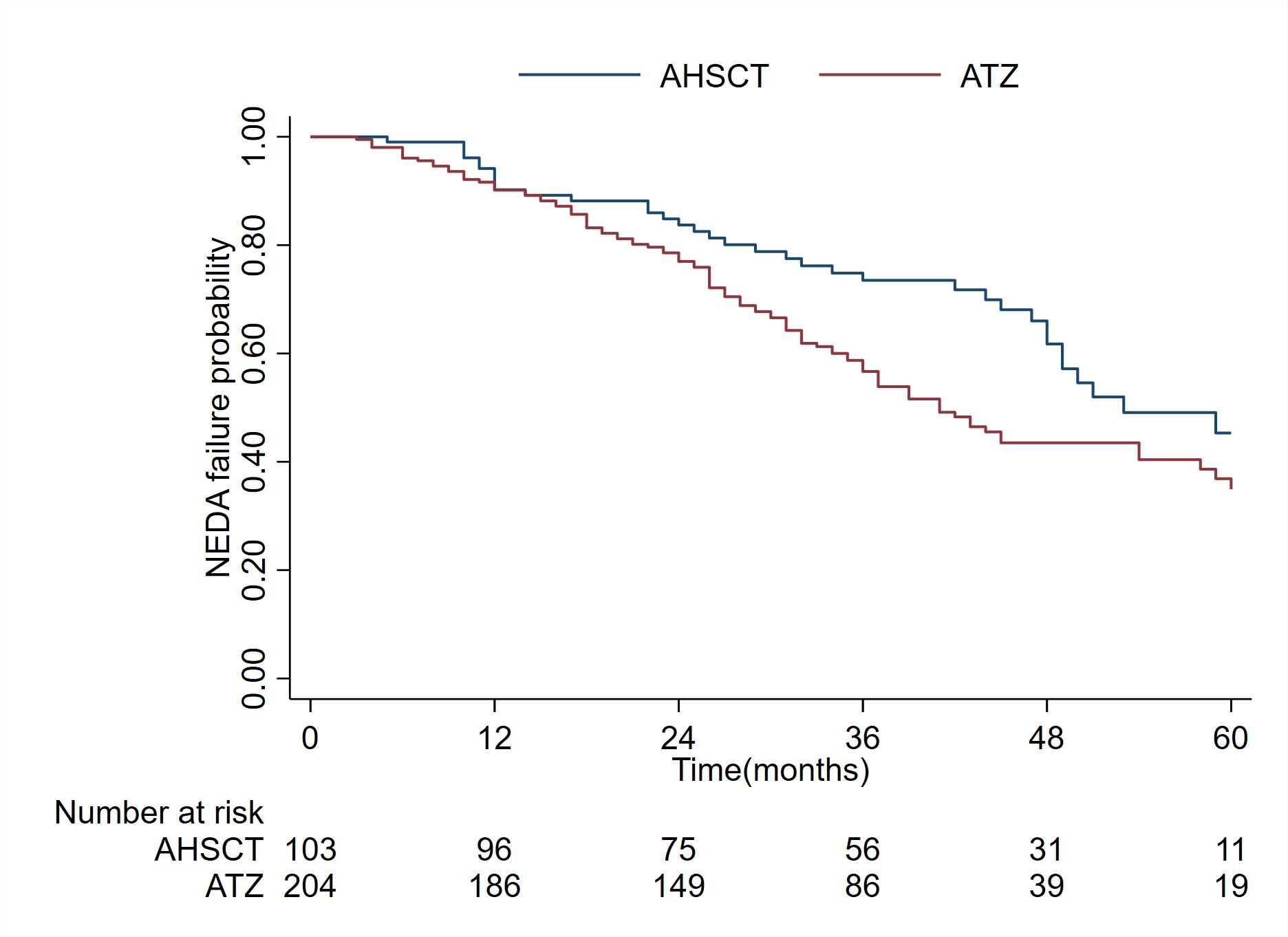


**Figure S2A. Comparative analysis between AHSCT and OCR; unweighted (from crude data) Kaplan Meier analysis. Cumulative probability of relapse.**


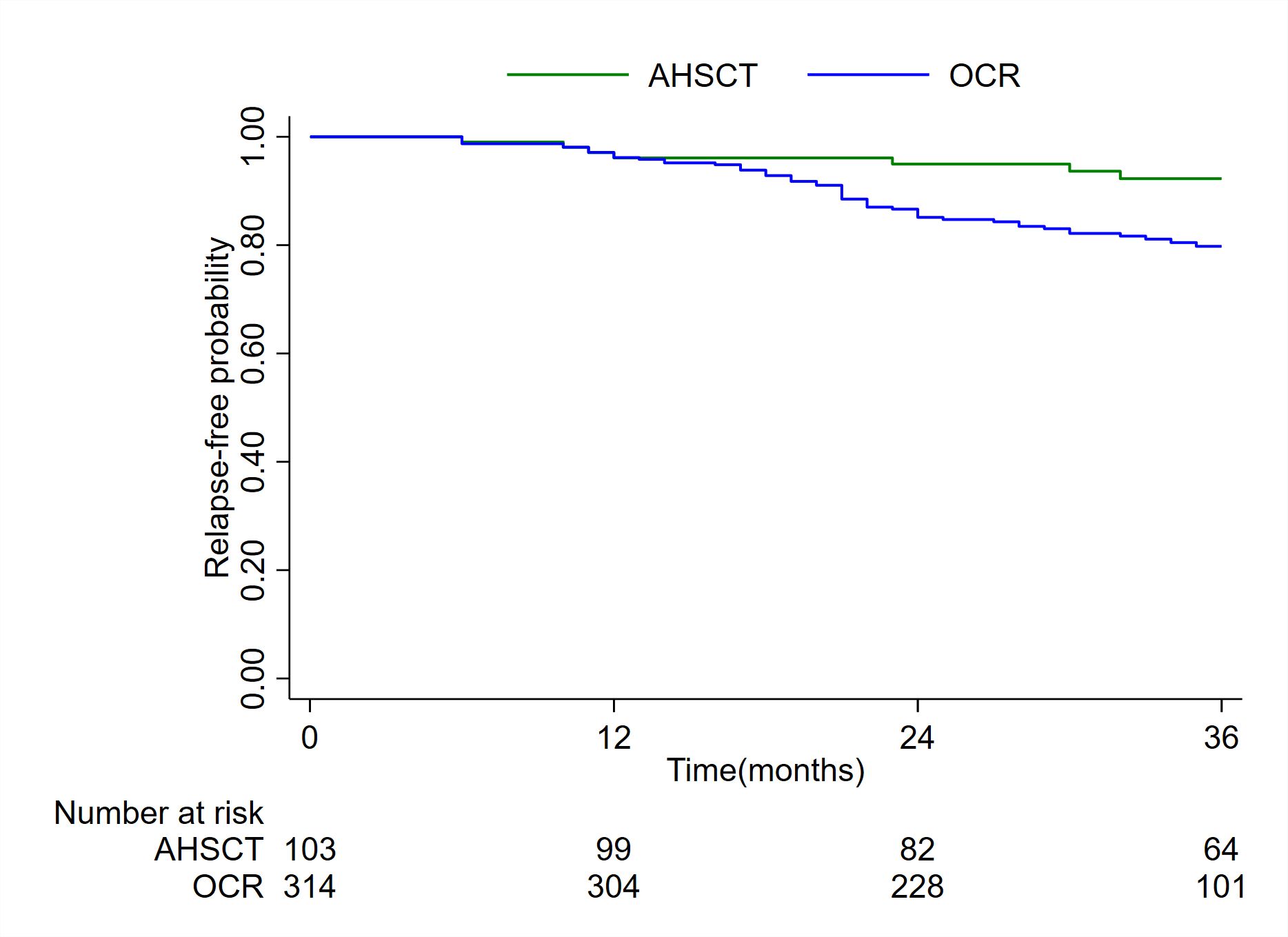


**Figure S2B. Comparative analysis between AHSCT and OCR; unweighted (from crude data) Kaplan Meier analysis. Cumulative probability of new MRI activity**


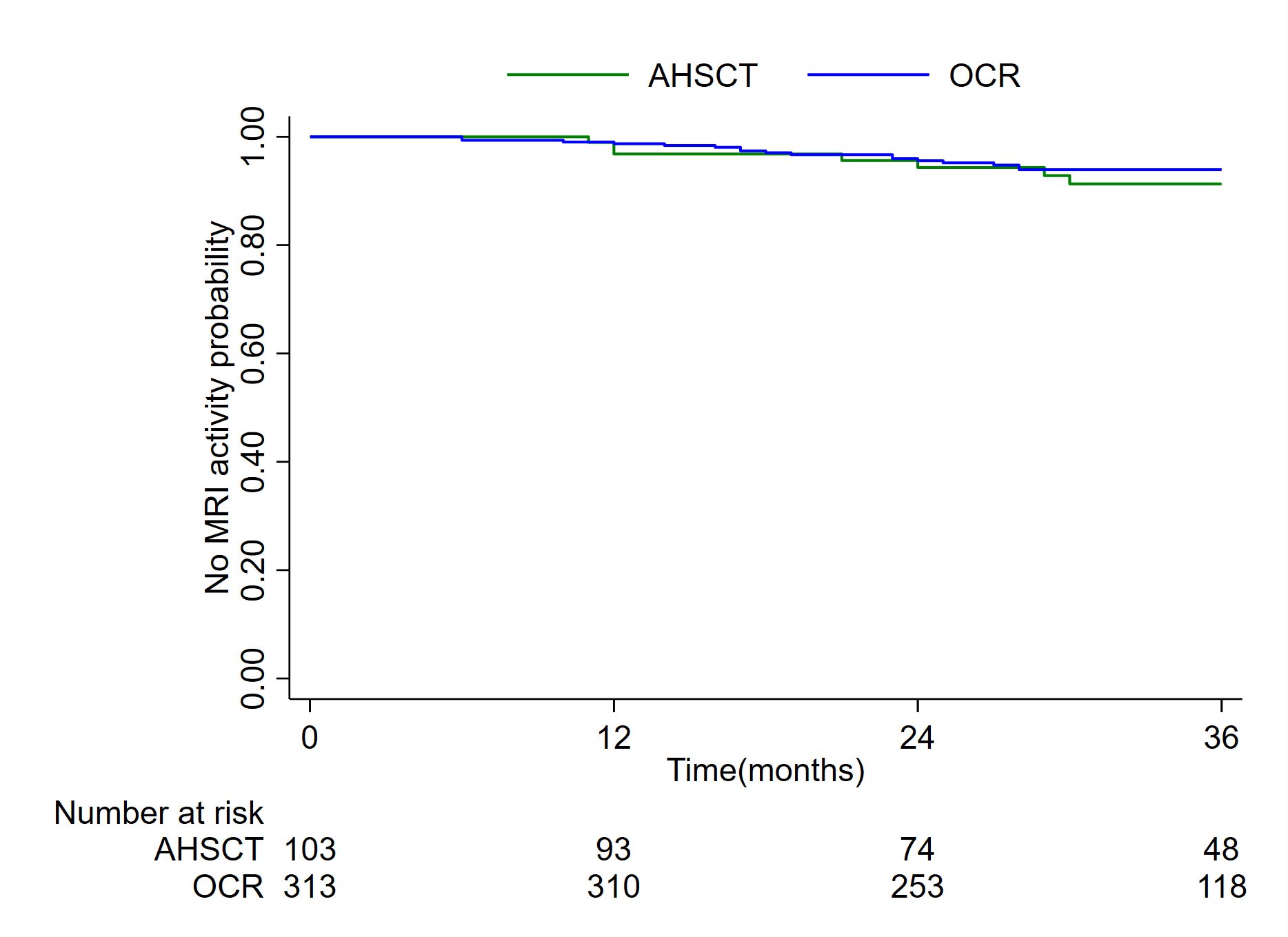


**Figure S2C. Comparative analysis between AHSCT and OCR; unweighted (from crude data) Kaplan Meier analysis. Cumulative probability of EDSS progression**

**
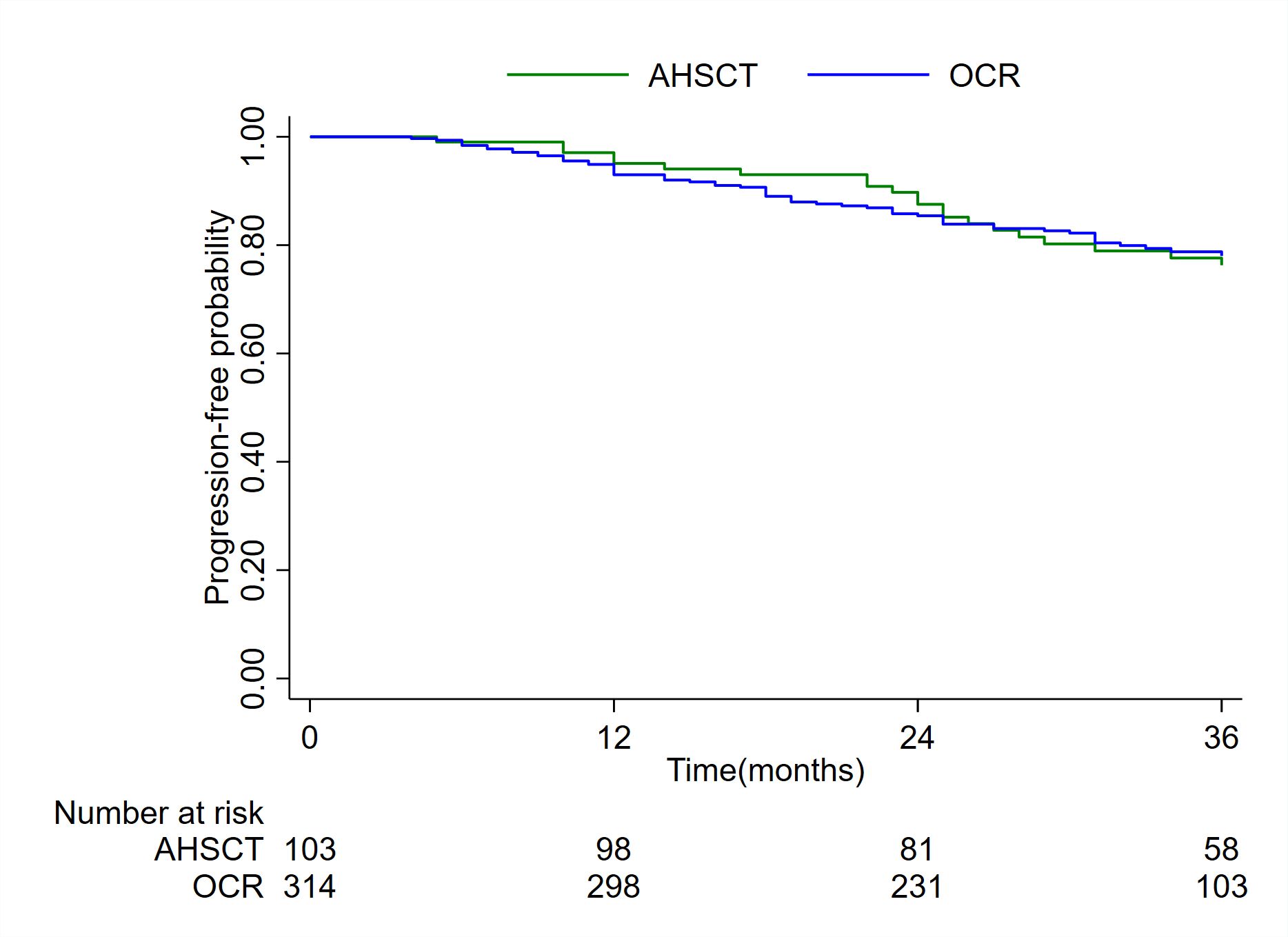
**

**Figure S2D. Comparative analysis between AHSCT and OCR; unweighted (from crude data) Kaplan Meier analysis. Cumulative probability of EDSS improvement.**

**
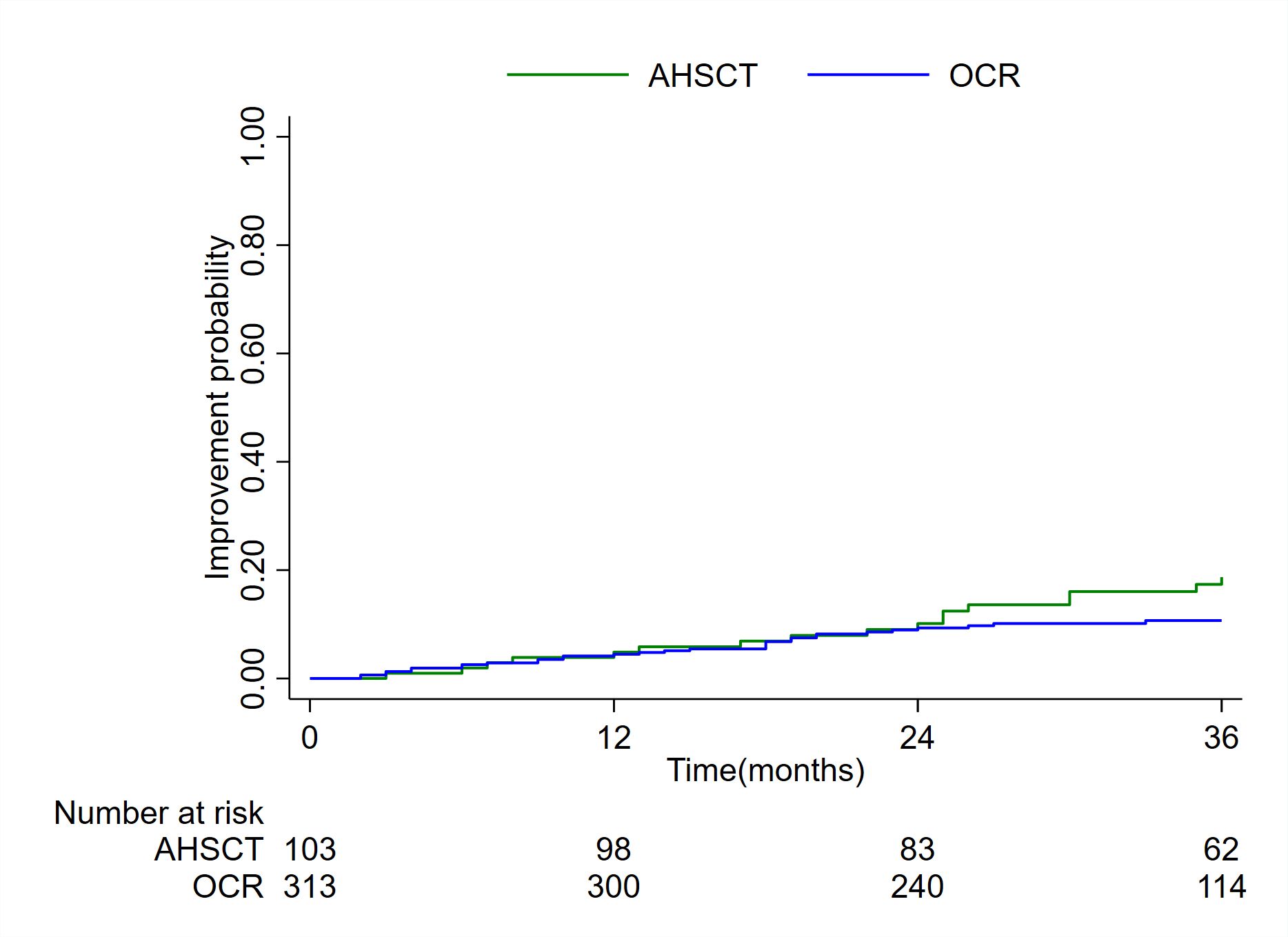
**

**Figure S2E. Comparative analysis between AHSCT and OCR; unweighted (from crude data) Kaplan Meier analysis. Cumulative probability of NEDA failure.**


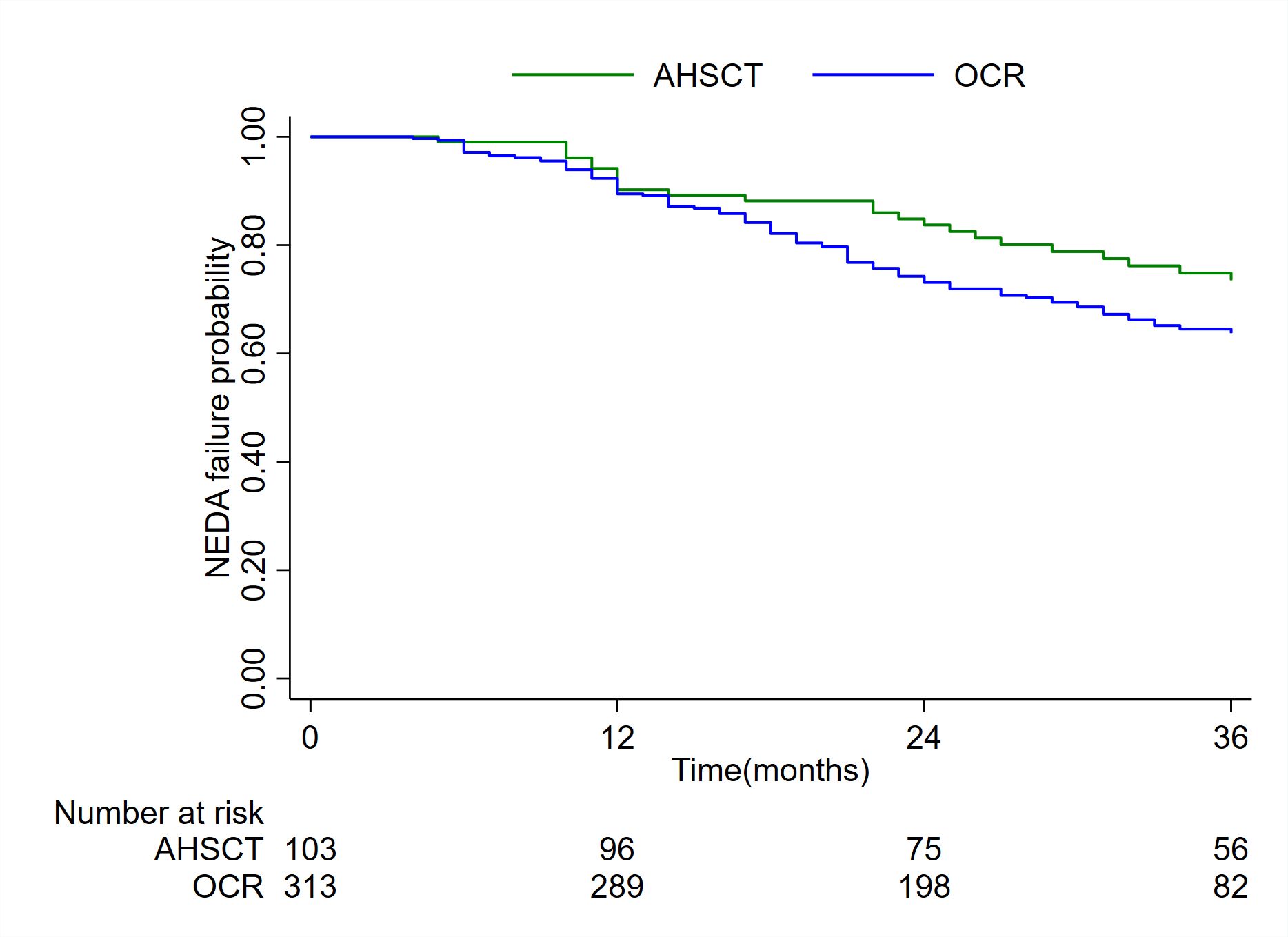

Supplement: Supplementary file 1 — DATA S1 Supporting Information. [file ANA-98-294-s001.docx]
